# Dynamic Modeling of Carbon Metabolism During the Dormant Period Accurately Predicts the Changes in Frost Hardiness in Walnut Trees *Juglans regia* L.

**DOI:** 10.3389/fpls.2018.01746

**Published:** 2018-12-05

**Authors:** Guillaume Charrier, André Lacointe, Thierry Améglio

**Affiliations:** INRA, PIAF, Université Clermont Auvergne, Clermont-Ferrand, France

**Keywords:** carbohydrates, dormancy, enzymatic activity, starch, tree physiology, winter biology

## Abstract

The leafless period is often considered as inactive, although trees have to actively modulate their metabolism through the cold acclimation/deacclimation processes, to cope with frost exposure during winter and to restore growth ability in spring. Carbon metabolism is a key component of these processes through the osmotic control of extracellular ice formation and the trophic control of bud growth. The influence of temperature on the inter-conversion between starch and soluble carbohydrate has been evidenced for years, but we are currently missing an operational tool to predict starch vs. soluble carbohydrate contents during this period, which should allow to better predict frost hardiness. For this purpose, we exposed 1-year-old branches of *Juglans regia* to constant temperature for one to 3 weeks and measured the changes in carbohydrate composition at three periods (autumn, winter, and spring). As expected, the temperature significantly affected the changes in carbohydrate composition, but the water content and the sampling period were also relevant. Higher starch hydrolysis was observed at low temperature (<5°C) for all sampling periods. Starch hydrolysis was also observed at warm temperature, but in autumn only. These data were used to compare three modeling approaches simulating the changes in carbohydrate composition through enzymatic analogy. The most empirical and the most mechanistic approach did not succeed to simulate external observations (Root Mean Standard Error of Prediction (RMSEP) > 30 mg.g DM^−1^, Efficiency (Eff) <0), whereas the intermediate model was more efficient (RMSEP = 15.19 mg.g DM^−1^, Eff = 0.205 and 16.61 mg.g DM^−1^, Eff = 0.366, for GFS (Glucose + Fructose + Sucrose) and starch, respectively). The accuracy of the model was further improved when using field data for calibration (RMSEP = 5.86 mg.g DM^−1^, Eff = 0.962; RMSEP = 10.56 mg.g DM^−1^, Eff = 0.752, for GFS and starch, respectively). This study provided an operative tool to simulate carbohydrate dynamics over leafless period that could predict frost hardiness with approx. 3.4°C accuracy with temperature, water content and initial starch and soluble carbohydrate measurements. It should now be tested under various meteorological conditions and biological systems.

## Introduction

In frost-exposed habitats (i.e., from temperate to boreal areas), perennial plants have to cope with freezing stress every winter. Successful transitions between growing seasons are therefore achieved through timely cold acclimation and subsequent deacclimation (Kalberer et al., 2006; Arora and Taulavuori, 2016). In parallel, bud growth is limited, first (in fall and winter) by endodormancy, then (in late winter and early spring) by ecodormancy, i.e., respective inhibition by intrinsic and environmental factors (mainly temperature; Lang et al., 1987). Trees are indeed cold acclimating during the endodormancy stage, then cold de-acclimating during the ecodormancy stage (Charrier et al., 2011). Despite the apparent ‘inactivity’ of plants and during a period where energy input is very limited or null, especially in deciduous species, cold acclimation is a complex process, involving numerous physiological mechanisms, prior to frost exposure (Pearce et al., 1996; Pearce, 2004; Charrier et al., 2015a).

Solute content (i.e., carbohydrates, organic or amino acids) increases and water content decreases in relation to frost hardiness in many perennial species (Reuther, 1971; Levitt, 1980; Guy, 1990; Gusta et al., 2004; Morin et al., 2007; Charrier et al., 2013a, 2015b). Different compounds, with contrasted efficiencies, have been observed across species, in relation to specific metabolism (Sakai, 1960, 1962) e.g., polyols (sorbitol or mannitol) in *Rosaceae* (e.g., *Sorbus aucuparia*; Sakai, 1966b; Raese et al., 1977; Ichiki and Yamaya, 1982, glucose, fructose and sucrose (GFS) in *Juglans sp* (Améglio et al., 2004; Poirier et al., 2010). Nitrogen metabolism may be involved as well resulting in amino acids (Smith, 1968) and proline accumulation (Withers and King, 1979). The intracellular concentration lowers the freezing temperature of the cytoplasm (*ca*. 1.86 K per mol solutes per kg water; (Cavender-Bares, 2005), which in turn favors ice nucleation to occur in the less concentrated apoplastic compartment. The ratio between GFS and water content thus accurately predict frost hardiness across seasons and organs in walnut trees, according to the osmo-hydric model (Charrier et al., 2013b). The extremely low water potential of ice (−1.16 MPa.K^−1^ below freezing temperature) pulls water molecules out through the membrane, further increasing solute concentration (Charra-Vaskou et al., 2015; Charrier et al., 2015b; Arora, 2018). Furthermore, solutes maintain a solvation layer thus protecting the surface of membranes and the macromolecules (Sakai, 1962; Heber and Santarius, 1973; Steponkus et al., 1977; Yoon et al., 1998, Kasuga et al., 2006, 2007).

Carbon metabolism exhibits significant changes throughout the leafless period (Sauter, 1988; Witt and Sauter, 1994; Lacointe et al., 1995; Wong et al., 2003; Poirier et al., 2010). Starch is almost totally hydrolyzed into osmotically active compounds from the end of the growing season through the mid-winter and re-synthesized during late winter and early spring (Siminovitch et al., 1953; Sakai, 1966a; Harms and Sauter, 1992; Sauter and Wellenkamp, 1998; Wong et al., 2003). Those carbohydrate dynamic changes can be related to changes in enzymatic activities such as α-amylase, β-amylase, sucrose synthase, sucrose phosphate synthase (Witt and Sauter, 1994; Schrader and Sauter, 2002). For instance, α-amylase and starch phosphorylase are activated by cold temperatures (Elle and Sauter, 2000). Furthermore, α-amylase is more tightly bound to starch grains at low temperature, optimizing starch hydrolysis (Witt and Sauter, 1995; Witt et al., 1995; Sauter et al., 1998). The expression in β-amylase is activated by both cold and warm temperatures (Kaplan and Guy, 2004). Starch conversion into soluble carbohydrates is consequently optimal at low temperatures (<10°C in poplar; Sauter, 1988). However, starch is also hydrolyzed at warmer temperature (i.e., >15°C) in *Salix sp*. (Sakai, 1966c) or *Juglans sp*. (Améglio et al., 2001; Charrier and Améglio, 2011). At the end of the winter, cells contain a low amount of starch but very high amount of soluble sugars.

Frost deacclimation is observed when temperature increases in late winter—early spring. According to Kalberer et al. (2006), this process is two-fold, including active and passive processes. Active deacclimation is induced by warm temperatures and involves structural and functional changes in relation to starch re-synthesis (Charrier et al., 2013a,b) and resumption of root water uptake (Pogosyan and Sakai, 1969). Passive deacclimation is a response to moderate warming (lower than 5K increase) via increased respiration, consuming cryoprotective solutes (Ögren, 1996). However, deacclimated plants are able to re-harden rapidly compared to the initial rate of hardening (2 days vs. 15 days, Chen and Li, 1980a,b). All enzymes involved in the starch hydrolytic pathway sharply decrease before or just after bud break resulting in starch re-synthesis (Witt and Sauter, 1994; Schrader and Sauter, 2002; Wong et al., 2003). After budbreak, soluble carbon compounds are transported within the xylem to carbon sinks (i.e., growing buds; Sauter, 1980, 1981, 1982; Sauter and Ambrosius, 1986; Améglio et al., 2000; Alves et al., 2001; Bonhomme et al., 2009). At this period, soluble carbohydrate supply the growth of buds and developing organs (i.e., leaves, roots and shoots; Marquat et al., 1999; Maurel et al., 2004). About one third of the reserves is used for the construction of the new leaves (Larcher, 1995). An accurately simulation of the carbohydrate metabolism is therefore crucial to assess frost hardiness along the frost-exposed period, and also to predict growth patterns after dormancy release.

Cold acclimation is controlled by environmental factors, mainly temperature and photoperiod (Aronsson, 1975; Christersson, 1978; Arora and Rowland, 2011; Pagter and Williams, 2011). Numerous models of cold acclimation, based on empirical relations between environmental factors and current frost hardiness level, have been developed so far (Fuchigami et al., 1982; Greer and Warrington, 1982; Leinonen, 1996; Jönsson et al., 2004; Ebel et al., 2005; Ferguson et al., 2011). However, although relevant in the current context, this cannot explain how a cold deprived tree would be able to harden (Schwarz, 1970; Alves et al., 2001; Charrier and Améglio, 2011). These observations suggest that starch to soluble carbohydrate conversion occurring at warm temperature (i.e., >15°C; Sakai, 1966c; Améglio et al., 2001) would induce cold acclimation (Charrier and Améglio, 2011). Despite the strong mechanistic link between osmotic compounds and frost hardiness, and the relation between environmental factors and synthesis of osmotic compounds, no process-based model has been developed so far that could predict frost hardiness (i.e., the highest temperature that would induce frost damages) in relation to carbohydrate metabolism and environmental factors, mainly the temperature. Previous studies reported an integrated response of frost hardiness to temperature averaged over several days and weeks (16 days in Poirier et al., 2010).

The present study aimed at constructing an operative model simulating the hydrolysis of starch into soluble *osmotica*, which would subsequently induce higher frost hardiness. We assumed that the dynamics of starch and soluble carbohydrates can be described by the catalytic activities under the control of phenological and thermal parameters. We examined the correlations between frost hardiness, carbohydrates, or water contents with temperature averaged on various periods (1–30 days) and selected one and three weeks delay to consider the integrated response. To feed the model with additional data, we thus stored walnut branches at constant temperature for 1–3 weeks at different periods, autumn (beginning of endodormancy stage), winter (transition between endo- and ecodormancy) and spring (middle of ecodormancy stage) and measured soluble carbohydrates and starch after storage. In comparison to initial conditions, we were able to calibrate a simple model of carbon metabolism that was confronted to field observations, which is an essential step toward process-based prediction of frost hardiness and trophic control of bud growth over the dormant period.

## Materials and Methods

### Plant Material

Mature European walnut trees (15–20 year-old *Juglans. regia* L. cv. Franquette) were grown in two different orchards in central France (low: 45° 46′ N 03°08′ E, 340 m a.s.l., and high elevation: 45°43′N 03° 01′ E 880 m a.s.l.). During five consecutive winters (2008–2012), one branch per tree (longer than 0.5 m) was harvested every month on the same five trees (natural conditions dataset NC, hereafter). During winter 2008–2009, 14 additional branches per tree were harvested for controlled-temperature treatment in autumn (22nd October 2008; beginning of endodormancy stage), winter (19th January 2009; transition between endo- and ecodormancy), and spring (17th March 2009; middle of ecodormancy stage) on five other trees (Year 1 from controlled conditions dataset CC, hereafter). During winter 2009–2010, 14 additional branches per tree were harvested for controlled-temperature treatment in autumn (4th November 2009), and 6 additional in winter (26th January 2010), and spring (30th March 2010) on five other trees (Year 2 from controlled conditions dataset, hereafter). To deplete the amount of non-structural carbohydrates (NSC), we almost completely defoliated three trees from the same variety (removing *ca*. 3,500 leaves and leaving only two terminal leaves for sap flow to be maintained) during the growing season (from 25th July 2009 until leaf fall).

### Controlled-Temperature Treatment

After sampling, one branch per tree was immediately used for carbohydrate and water content measurement (t_0_). To take into account any memory effect (i.e., the effect of the previous climatic history of the tree on physiological variables) and select an optimal treatment period, thermal variables (i.e., minimum, maximum, and average temperature) were averaged over 0 (i.e., current) to 30 days with 1 day increment (Table S1). Given the correlations between physiological and thermal variables, samples were stored for one to three weeks (see results). The basal cut ends of other branches from the CC dataset were sealed with wax and stored at constant temperature, humidity and light for seven (t_7_) and 19–21 days. During year 1, branches were stored at different temperatures i.e., −3, +1, +5, +10, +15, +20, and +25°C with constant photoperiod (i.e., Autumn: 10 h light/14 h night; Winter: 9 h light/15 h night and Spring: 12 h light/12 h night; see Table S2 for a detailed description of the different treatments). The second year, the complete set of temperature was repeated during autumn, but only three temperatures were tested during winter and spring: −3, +15, and +25°C.

### Frost Hardiness Test

For the NC dataset, sampled branches from each individual were cut into six 5-cm long segments and exposed to four different freezing temperatures among this set of temperatures: −5, −10, −15, −20, −30°C, −40°C, then thawed to assess frost hardiness (FH) using the electrolyte leakage method. Depending on the season, either the highest or the lowest temperatures were not used. Two supplementary subsamples were exposed to control temperature (+5°C) and maximal freezing temperature (*ca*. −75°C). Freezing and thawing rates were set to 5 K.h^−1^. Supplementary details are provided in Charrier and Améglio (2011).

### Water Content

Small pieces between segments used for FH tests were sampled along the branch. Fresh matter weights (FM) were measured, then the samples were frozen with liquid nitrogen. After freeze-drying, dry matter weights (DM) were measured and water content (WC) was calculated as:

(1) $$\begin{array}{l} WC=\;\frac{\left(FM-DM\right)}{DM} \end{array}$$

### Carbohydrate Extraction and Quantification

Lyophilized samples (*m* > 2 g) were ground to a powder that was used (50 mg) to extract and measure the soluble carbohydrate content using HPLC method. Starch content was measured using enzymatic method. Details are provided in Charrier and Améglio (2011).

### Carbohydrate Metabolism Modeling

#### Description of the Model

Change in carbohydrate composition was simulated as the result of different chemical reactions through the winter dynamics of related enzymatic activities impacting three functional components: reserve (i.e., starch), soluble carbohydrates (i.e., GFS representing approx. 90% of soluble carbohydrates in *Juglans sp*), and respiration (temperature-dependent but unregulated CO_2_ efflux). Chemical reactions were limited by enzymatic activities (i.e., acting as limiting factors). Slower molecular movements inducing fewer collisions between substrates and enzymes (lower temperatures) and higher protein denaturation (higher temperatures) alter the enzymatic reaction rates. Enzymatic activities were therefore optimal at a given temperature (μ_i_). Although the slope at low and high temperatures may be different, we chose a symmetric Gaussian function to limit the number of parameters of the model. As observed in the literature (Sakai, 1966c), starch hydrolysis was considered under the dependence of two enzymatic activities with contrasted optimal temperatures i.e., cold (k_1c_) and mild temperatures (k_1m_), whereas starch re-synthesis (k_2_) and respiration (R) were assumed depending each on a single reaction:

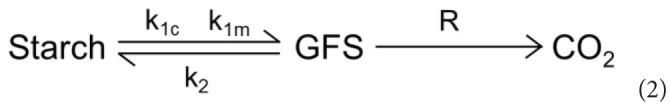



The enzymatic activity was considered under the dependency of the phenological stage *PS* divided into two different phases: endodormancy and ecodormancy, which occur sequentially (Hänninen and Kramer, 2007). Endodormancy is released by chilling temperatures (Lang et al., 1987). The cumulative action of chilling temperatures is quantified as “Chilling Units,” *CU(t)*, according to the inverse of the Richardson function as defined in (Chuine et al., 2016) (Equation 3) with a zero starting value.

(3) $$\begin{array}{l} CU\left(t+1\right)=CU\left(t\right)\;+\text{ Max}\left(\text{Min}\left({\text{T}}_{\text{high}}-\;\theta \left(t\right);\;{\text{T}}_{\text{high}}-{\text{T}}_{\text{low}}\right);0\right) \end{array}$$

with *CU*(*t*), the cumulated chilling units at day *t*, T_high_ the temperature threshold above which the daily mean temperature θ(*t*) has no chilling effect, and T_low_ the temperature threshold below which the chilling effect is constant (linearly decreasing over the [T_low_; T_high_] range).

When *CU*(*t*) reaches the threshold value CU_crit_ (see Table 1 for parameter values), endodormancy is released and ecodormancy stage initiates. The cumulative effect of mild temperatures on within-bud growth, i.e., ecodormancy status, was quantified as “Forcing Units” *FU(t)*, modeled as a sigmoid function (Equation 4, Caffarra et al., 2011).

(4) $$\begin{array}{l} FU\left(t+1\right)=FU\left(t\right)+\frac{1}{1+e^{-\text{slp}\left(\;\theta \left(t\right)-{\text{T}}_{50}\right)}} \end{array}$$

with *FU*(*t*), the cumulated forcing units at day t, slp (−0.244°C^−1^), the slope of the function at the temperature inducing half of the maximal apparent growth rate T_50_ (13.46°C).

**Table 1:** Parameters for the phenological model.

**Sub-model**	**Description**	**Value**	**Source**
**ENDODORMACY RELEASE**			
T_0_ (DOY)	Beginning of endodormancy	244	(Chuine et al., 2016)
T_low_ (°C)	Threshold temperature below which CU is maximum	3.1	(Chuine et al., 2016)
T_high_ (°C)	Threshold temperature above which CU is null	26.7	(Chuine et al., 2016)
CU_crit_ (CU)	Amount of Chilling Units to complete endodormancy stage	2298.8	(Chuine et al., 2016)
**ECODORMANCY RELEASE**			
Slp	Slope at the inflection point T_50_	0.244	(Charrier et al., 2011)
T_50_ (°C)	Temperature inducing half of the maximal apparent growth rate	13.5	(Charrier et al., 2011)
FU_crit_ (FU)	Amount of Forcing Units to complete the ecodormancy stage	21.2	(Charrier et al., 2011)

When *FU(t)* reaches the value FU_crit_, endormancy is over with bud breaking. This lead to the definition of a phenological stage (*PS)* as the ratio between thermal time (i.e., Chilling *CU* and Forcing Units *FU* for endo- and ecodormancy, respectively) and thermal requirements (CU_crit_ and FU_crit_ for endo- and ecodormancy, respectively). The parameters used to describe the influence of environmental parameters on dormancy status (endo- and ecodormancy release) and to compute CU, FU and PS were fixed according to the literature (Charrier et al., 2011, 2018; Chuine et al., 2016). The model assumes a sequential transition between endo- and ecodormancy and PS is an index ranging from 0 (beginning of endodormancy) to 2 (budburst), with 1 being the transition between endo- and ecodormancy:

(5) $$\begin{array}{l} PS=\frac{CU}{\text{C}{\text{U}}_{\text{crit}}}+\frac{FU}{\text{F}{\text{U}}_{\text{crit}}} \end{array}$$

Considering the effect of annual cycle and temperature on enzymatic activities, the catalytic rates (k_i_; i = 1c, 1m or 2) depended on temperature (θ, in °C) and phenological stage (PS) according to a combination of linear and Gaussian functions:

(6) $$\begin{array}{l} k_{i}=\max\left[0;\left({\text{a}}_{\text{i}}\cdot PS+{\text{b}}_{\text{i}}\right)\cdot \frac{e^{-{\left(\theta -\mu_{\text{i}}\right)}^{2}}}{\sigma_{i}\cdot \sqrt{2\pi }}\right] \end{array}$$

where a_i_ and b_i_ are coefficients that modulate enzymatic activity with phenological stage, θ is the mean temperature of the day, μ_i_ is the optimal temperature for maximal activity, and σ_i_ is the standard deviation.

Significant changes are expected to occur in the catalytic rate during the transition from endodormancy to ecodormancy, a_i_ and b_i_ are therefore assigned different values for *PS* lower *vs*. higher than 1 (a_i endo_, a_i eco_, b_i endo_, and b_i eco_). However, to prevent sharp changes in the carbon metabolism at the transition between endo- and ecodormancy, the catalytic rate at this time, k_itrans_, was defined as follows:

(7) $$\begin{array}{l} {\text{k}}_{\text{i trans}}={\text{a}}_{\text{i endo}}+{\text{b}}_{\text{i endo}}\;={\text{a}}_{\text{i eco}}+{\text{b}}_{\text{i eco}} \end{array}$$

with a_i endo_ = (k_i trans_ + d_i endo_)/2, b_i endo_ = (k_i trans_ - d_i endo_)/2, a_i eco_ = (k_i trans_ + d_i eco_)/2, b_i eco_ = (k_i trans_ + d_i eco_)/2, to reduce the number of parameters to fit (d_i_ being the difference between a_i_ and b_i_).

The respiration rate was modulated by the current water content (assuming linear variation between two successive sampling dates) and temperature but not substrate concentration (k_3_; order 0).

(8) $$\begin{array}{l} R=\frac{{\text{R}}_{\text{Max}}}{1+{\text{exp}}^{a_{3}}\cdot \left(WC-{\text{b}}_{3}\right)}\cdot {{\text{Q}}_{10}}^{\left(\frac{\theta -{\text{T}}_{\text{ref}}}{10}\right)} \end{array}$$

where a_3_ and b_3_ are coefficients that modulate respiration in relation with water content, R_Max_ is the maximum respiration rate at T_ref_, θ is the mean temperature of the day, Q_10_ is the temperature coefficient and T_ref_ is a reference temperature (i.e., 15°C).

GFS contents were calculated at a daily scale, with distinct optimal temperature (μ_i_) for the different catalytic rates using air temperature and water content at the daily scale and initial GFS and Starch contents as inputs. The different formalisms, along the *continuum* from empirical to mechanistic, were compared.

The direct effect of the temperature on catalytic activity was simulated by the simple model:

(9) $$\begin{array}{l} GFS\left(t+1\right)=GFS\left(t\right)+k_{1c}\left(t\right)+k_{1m}\left(t\right)-k_{2}\left(t\right)-R\left(t\right) \end{array}$$

As the observed variability in carbohydrate composition was reduced after normalization of the daily change in GFS and starch by the initial (i.e., at t_0_) amount of starch and GFS, respectively (**Figures 3A–F**; Figures S1–S3; Tables S4–S8), we developed the intermediate model based on the mechanistic assumption that the change in the amount of the product of an enzymatic reaction can be limited by the amount of substrate:

(10) $$\begin{array}{l} GFS\left(t+1\right)=GFS\left(t\right)+\left[k_{1c}\left(t\right)+k_{1m}\left(t\right)\right]\cdot Starch\left(t\right)\\ \;-\;k_{2}\left(t\right)\cdot GFS\left(t\right)-R\left(t\right) \end{array}$$

where k_1c_ + k_1m_ and k_2_ correspond to the starch hydrolyzing activity and re-synthesis, respectively.

According to Michaelis-Menten kinetics, stating that the product formation ultimately depended on the catalytic activity (k_i_), the enzyme ([E]) and substrate concentrations ([S]) and the Michaelis constant (K_M_), with *v*_*Max*_ = *k*_*i*_·[*E*], we developed the complete model:

(11) $$\begin{array}{l} v_{i}=\frac{v_{Max}}{K_{Mi}+\left[S\right]}\cdot \left[S\right] \end{array}$$

(12) $$\begin{array}{lll} GFS\left(t+1\right)=GFS\left(t\right) & + & \left[\frac{v_{Max1c}\left(t\right)}{{\text{K}}_{\text{M}1\text{c}}+Starch\left(t\right)}\;+\;\frac{v_{Max1m}\left(t\right)}{{\text{K}}_{\text{M}1\text{m}}+Starch\left(t\right)}\right]\cdot \\ Starch\left(t\right)-\frac{v_{Max2}\left(t\right)}{{\text{K}}_{\text{M}2}+GFS\left(t\right)}\cdot GFS\left(t\right)-R\left(t\right) \end{array}$$

where $\frac{v_{Max1c}\left(t\right)}{{\text{K}}_{\text{M}1\text{c}}+Starch\left(t\right)}+\frac{v_{Max1m}\left(t\right)}{{\text{K}}_{\text{M}1\text{m}}+Starch\left(t\right)}$ and $\frac{v_{Max2}\left(t\right)}{{\text{K}}_{\text{M}2}+GFS\left(t\right)}$ correspond to the starch hydrolyzing activity and re-synthesis, respectively.

For all models, starch content can be derived from the following equation:

(13) $$\begin{array}{l} Starch\left(t+1\right)=Starch\left(t\right)-\left(GFS\left(t+1\right)-GFS\left(t\right)\right)-R\left(t\right) \end{array}$$

Finally, the ability of the model to predict frost hardiness (FH) was tested via the unified osmo-hydric model developed by Charrier et al. (2013a). The depression of the freezing point in cells favors the liquid water to crystallize in the apoplastic compartment, subsequently leading to cellular dehydration and further concentration. This model, which was developed across various organs (from fine roots to buds) and tissues (bark, and xylem) of walnut trees *J. regia* describes the depression of the freezing point and related FH through the nonlinear interaction between GFS (predicted by the carbon metabolism model) and WC (fixed according to observations).

(14) $$\begin{array}{l} FH=\text{a}\cdot \frac{Ln\left(GFS\right)}{WC}+\text{b} \end{array}$$

#### Optimization of the Parameters

Water content was fixed according to the observations with linear interpolation between two observations. The sets of parameters (*n* = 19, 19, and 22, for simple, intermediate and complex, respectively; Table S3) were calibrated (i) using the data from the CC dataset and compared to NC dataset as a validation, or (ii) splitting the NC dataset into two separate datasets for calibration (NC_1_) and validation (NC_2_). The optimization was performed by minimizing the residual sum of square between simulated values and measured data of starch and GFS using the Nelder Mead algorithm (package *nloptr* in R). The algorithm was run up to 200,000 times until convergence (relative tolerance = 10^−8^) starting from 100 sets of initial values distributed within a realistic range of the values for each parameter, according to an optimized Latin Hypercube Sample to avoid ending in a local minimum (package *lhs*).

### Statistical Analysis

The correlations between physiological (frost hardiness, starch, GFS and water contents) and meteorological variables from the closest weather stations (*ca*. 2 km away from the orchards) were measured using Pearson's product-moment coefficient (ρ*)*.

The performance of each model was estimated via different indexes:

(15) $$\begin{array}{l} \text{Efficiency}:Eff=\frac{\left(SS_{tot}-SS_{res}\right)}{SS_{tot}} \end{array}$$

where SS_tot_ and SS_res_ are the total and residual sums of square, respectively.

(16) $$\begin{array}{l} \text{Root Mean Standard Error}:\;RMSE=\sqrt{\frac{SS_{res}}{n}} \end{array}$$

where n is the number of observations.

## Results

### Correlation Between Physiological and Thermal Variables

**Figure 1:**
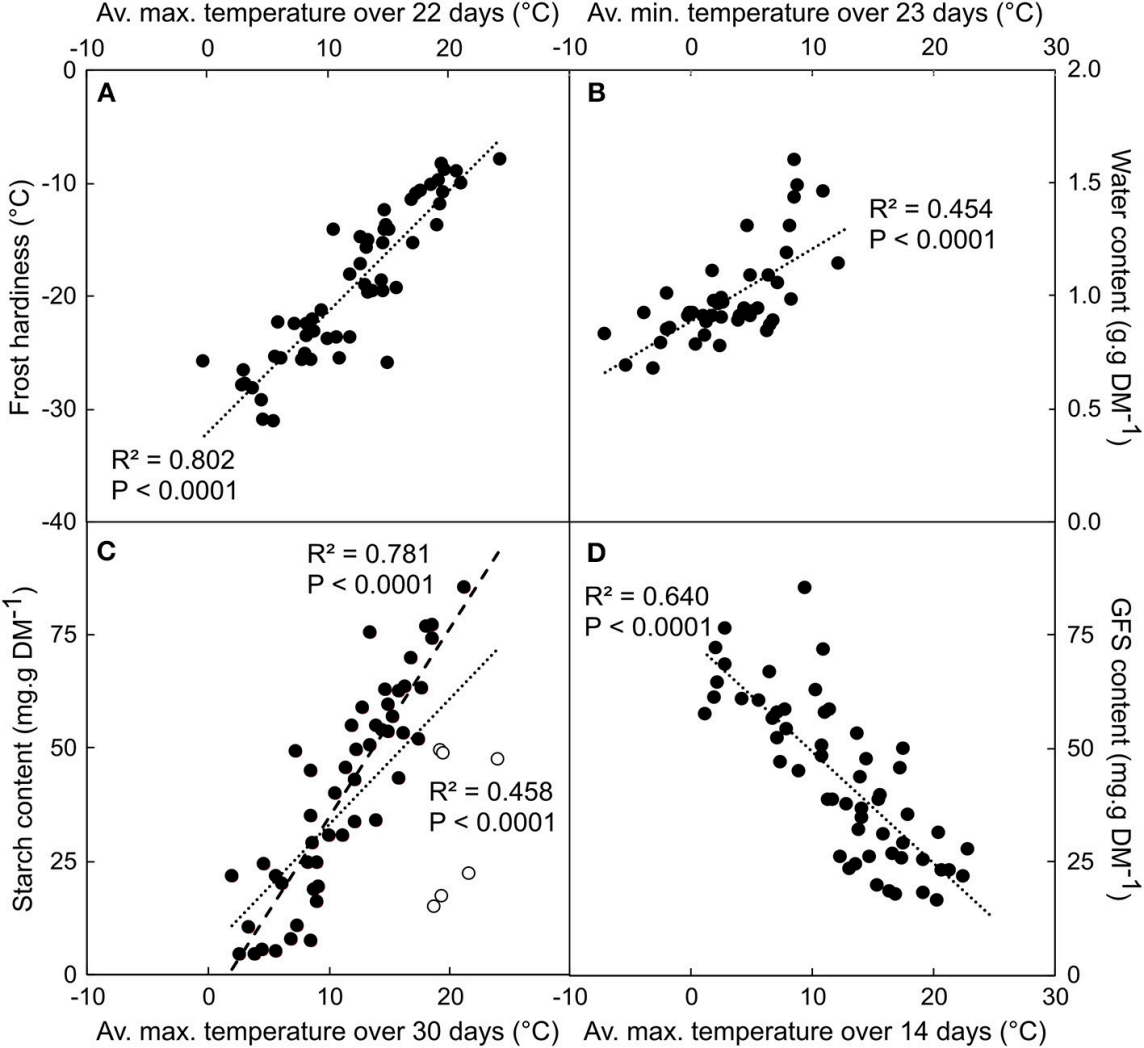
Correlation between frost hardiness **(A)**, water **(B)**, starch **(C)**, and GFS **(D)** contents depending on average maximum (or minimal for water content) temperature calculated over 22 (FH), 14 (GFS), 30 (Starch), and 23 (WC) days period. Data were collected during six different winter dynamics from 2007 until 2012. Each point represents the mean of *n* = 5 replicates. Open circles in **(C)** represent data collected during the leafy period that were included or not in the linear regression (dotted and dashed line, respectively).

The correlation between physiological (GFS, starch, frost hardiness and water content) and thermal (minimum, average and maximum temperature averaged over the last 1–30 days before harvest) variables were all highly significant (*P* < 0.0005; Table S1). However, the correlation coefficient (ρ) was slightly higher for maximum than mean or minimum temperature, except for water content, for which minimum temperature was better correlated. Over the last 7 days before sampling (from day 0 to 7), ρ increased with the number of days integrated in the calculation and became relatively stable over a longer period. The highest ρ was observed for frost hardiness *vs*. average maximal temperature averaged over 22 days (ρ = 0.896; *P* < 0.0001; Figure 1A). A similar period (23 day-long) was observed for highest ρ between water content and minimal temperature (ρ = 0.674; *P* < 0.0001; Figure 1B). Soluble carbohydrates and starch contents exhibited stronger correlations with maximal temperature calculated over *ca*. two weeks: GFS over 14 days (ρ = −0.800; *P* < 0.0001; Figure 1C) and starch over 15 days (ρ = 0.681; *P* < 0.0001; Figure 1D). However, when removing the observations from the leafy period (i.e., before leaf fall, or after budburst, as presented in open symbols in Figure 1D), much stronger correlation was observed between starch content and temperature averaged over 30 day-long periods (ρ = 0.884; *P* < 0.0001).

### Controlled-Temperature Treatment and Changes in Non-Structural Carbohydrates

**Figure 2:**
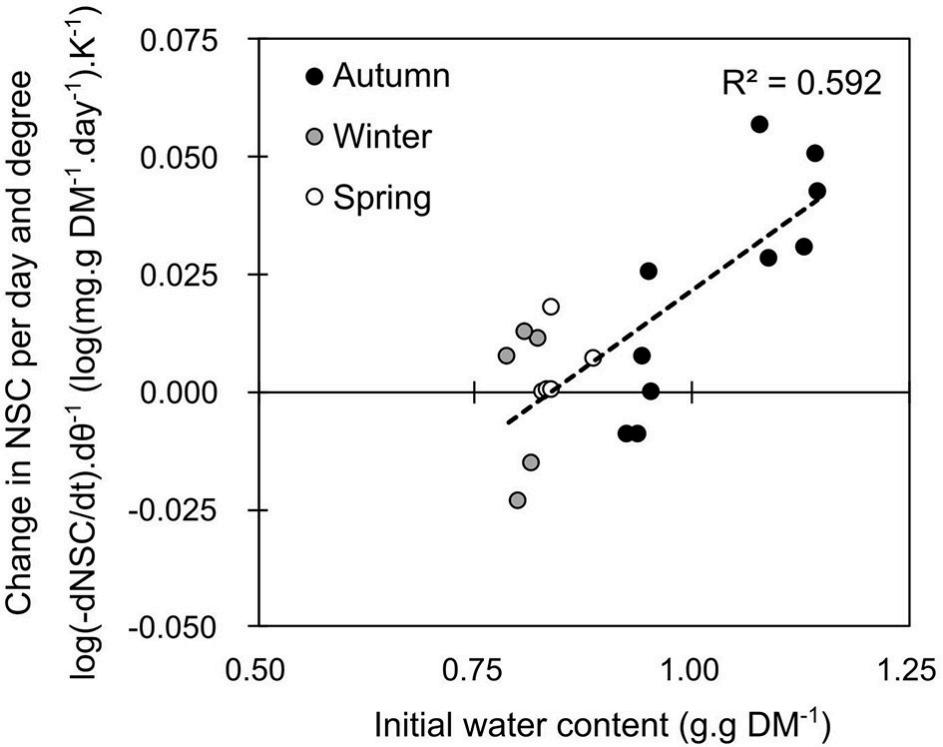
Average daily loss in total non-structural carbohydrates (log(dNSC) per Kelvin) over 7 days of constant temperature treatment depending on initial water content. Each point represents the estimate of the slope from seven branches of an individual tree exposed to seven different temperature from −3 to +25°C.

According to the correlations described above, branches that were sampled at different seasons were exposed to constant temperature for one to three weeks. The total amount of NSC within a branch was significantly affected by temperature, but differences related to the period of sampling also appeared. Daily losses in total non-structural carbohydrates (δNSC per day) were higher in autumn (−2.38 ± 0.21 mg.g DM^−1^.day−1; mean ± SE, *data not shown*) than in spring (−1.08 ± 0.20 mg.g DM^−1^.day−1, *data not shown*) and winter (−0.28 ± 0.12 mg.g DM^−1^.day^−1^, *data not shown*). Warmer temperature increased the loss in total non-structural carbohydrate content during autumn (*P* = 0.031), but not in spring (*P* = 0.280) nor winter (*P* = 0.486). The logarithmic variation in NSC per day [log(δNSC.day−1)] was significantly correlated with temperature (*P* = 0.0233) and with the interaction between temperature and initial water content of the branch (*P* = 0.0135; Table S4). Accordingly, the variation in NSC with respect to temperature [i.e., expressed as the slope of the regression between the logarithmic variation in NSC per day and the temperature: log(δNSC.day−1.K−1)] was significantly correlated with the initial water content of the branch (*P* = 0.005; Figure 2). Higher respiratory losses with warmer temperature were indeed recorded when water content was higher than one.

**Figure 3:**
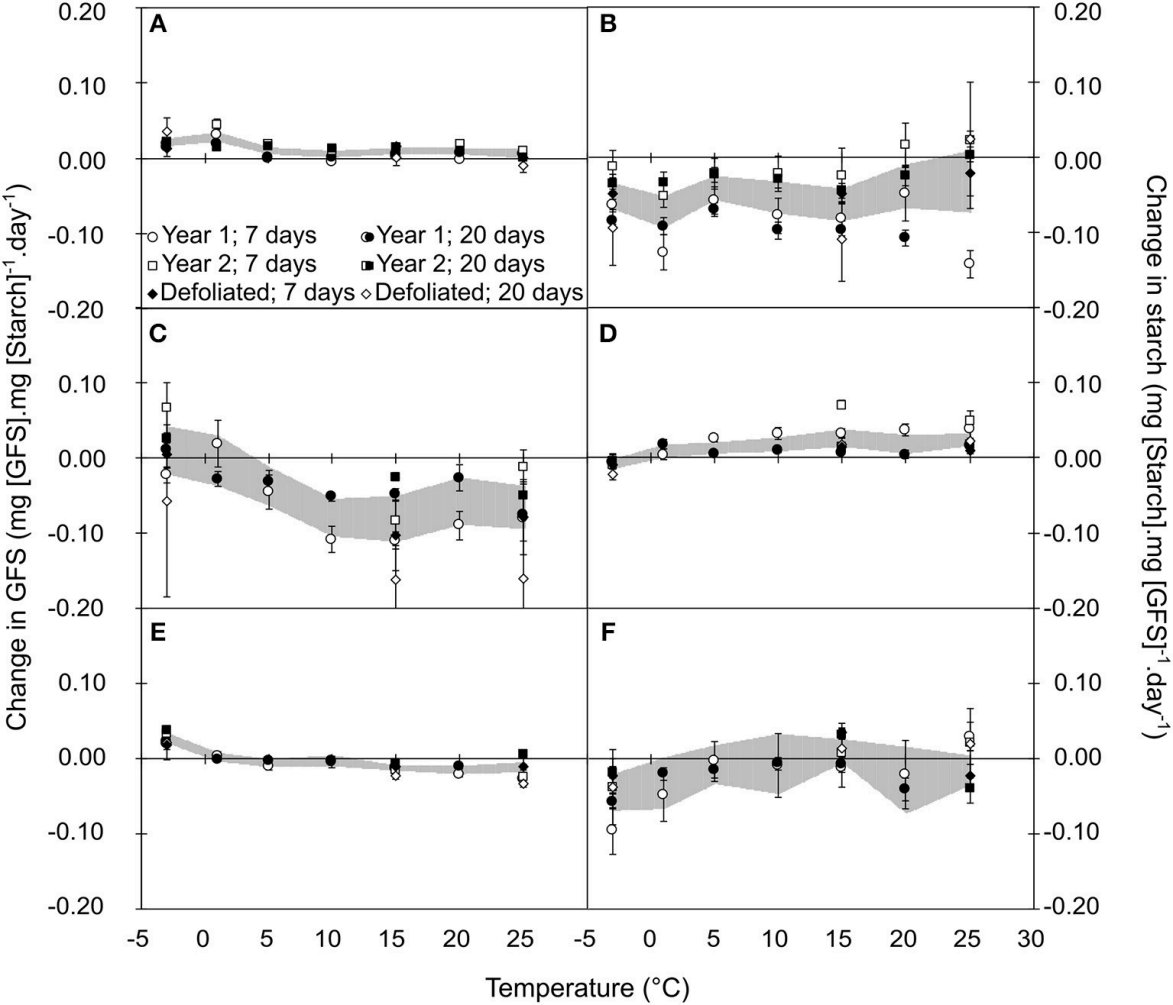
Relative variation in GFS **(A,C,E)** and Starch **(B,D,F)** normalized by Starch and GFS, respectively, after 7 (open symbols) and 20 days (closed symbols) of constant temperature treatment during autumn **(A,B)**, winter **(C,D)**, and spring **(E,F)**. Each point represents the mean and standard error of *n* = 5 replicates (year 1 and 2) or *n* = 3 replicates (defoliated trees). Shaded areas represent the confidence interval at 95% for the bulk of six cases, to indicate the general trend.

**Figure 4:**
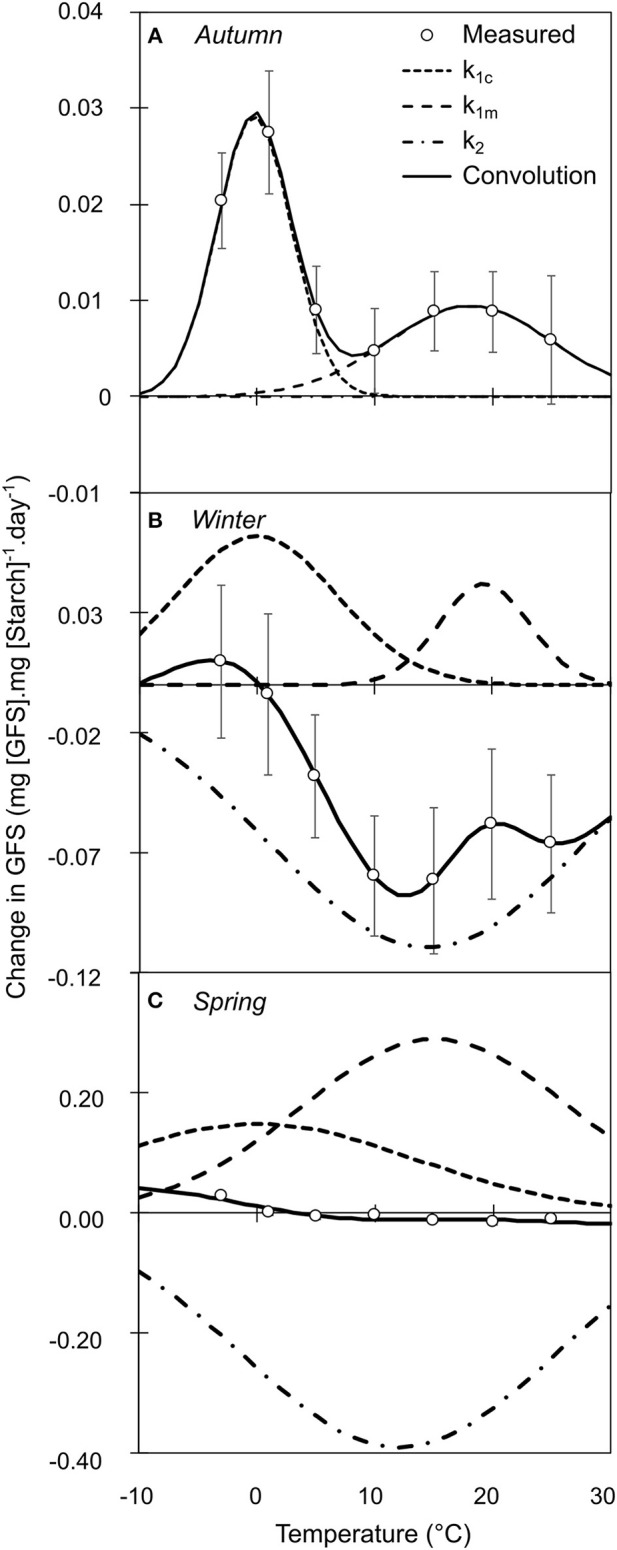
Relative variation in GFS normalized by initial Starch content after constant temperature treatment during autumn **(A)**, winter **(B)**, and spring **(C)**. Each point represents the mean and standard error of *n* = 26 replicates, across the 2 years of experiment. The black line represents the convolution of three distinct sigmoid functions (k_1c_: short dash; k_1w_: long dash and k_2_: dash-and-dot). The elementary sigmoid functions were relatively constant for optimal temperature, although their maximal value varied over winter.

Qualitative composition of NSCs varied in relation to temperature, season, year and treatment (Tables S5–S7). Based on the mechanistic assumption that the change in the amount of the product of an enzymatic reaction can be limited by the amount of substrate, we normalized the daily change in GFS and starch by the initial amount of starch and GFS, respectively. For illustrative purpose, we fitted the variation in GFS normalized by starch content as the convolution of three different Gaussian functions, two positive representing the cold and warm active hydrolysis and one negative representing the starch re-synthesis (**Figure 4**). In autumn, GFS increased and starch decreased for all temperatures with two peak values at 0 and +15°C, whereas lower changes were observed at *ca*. +10°C (Figures 3A,B). During winter, GFS increase was observed at low temperature (<0°C), whereas a decrease in GFS and increase in starch was observed at warmer temperature (Figures 3C,D). During spring, GFS only increased for temperature lower than 1°C, whereas starch was relatively stable (Figures 3E,F). Finally, low temperature (< 0°C) induced an increase in GFS content (i.e., promoting starch hydrolysis into GFS) for all the different periods (*P* = 0.2646; Figure 4). At warm temperature (*ca*. 17.5°C), slightly higher GFS content was observed than at colder temperature (Figures 4A,B). GFS decrease, and presumably starch re-synthesis, was observed during winter and spring at mild temperature (> 8°C). Despite very similar optimal temperature for the different catalytic activity (cold hydrolysis: −0.27 < μ_1c_ < 0°C; mild hydrolysis: 15.12 < μ_1m_ < 19.33°C and starch re-synthesis: 12.00 < μ_1c_ < 14.49°C; Figure 4), the catalytic rate varied along the leafless period.

**Figure 5:**
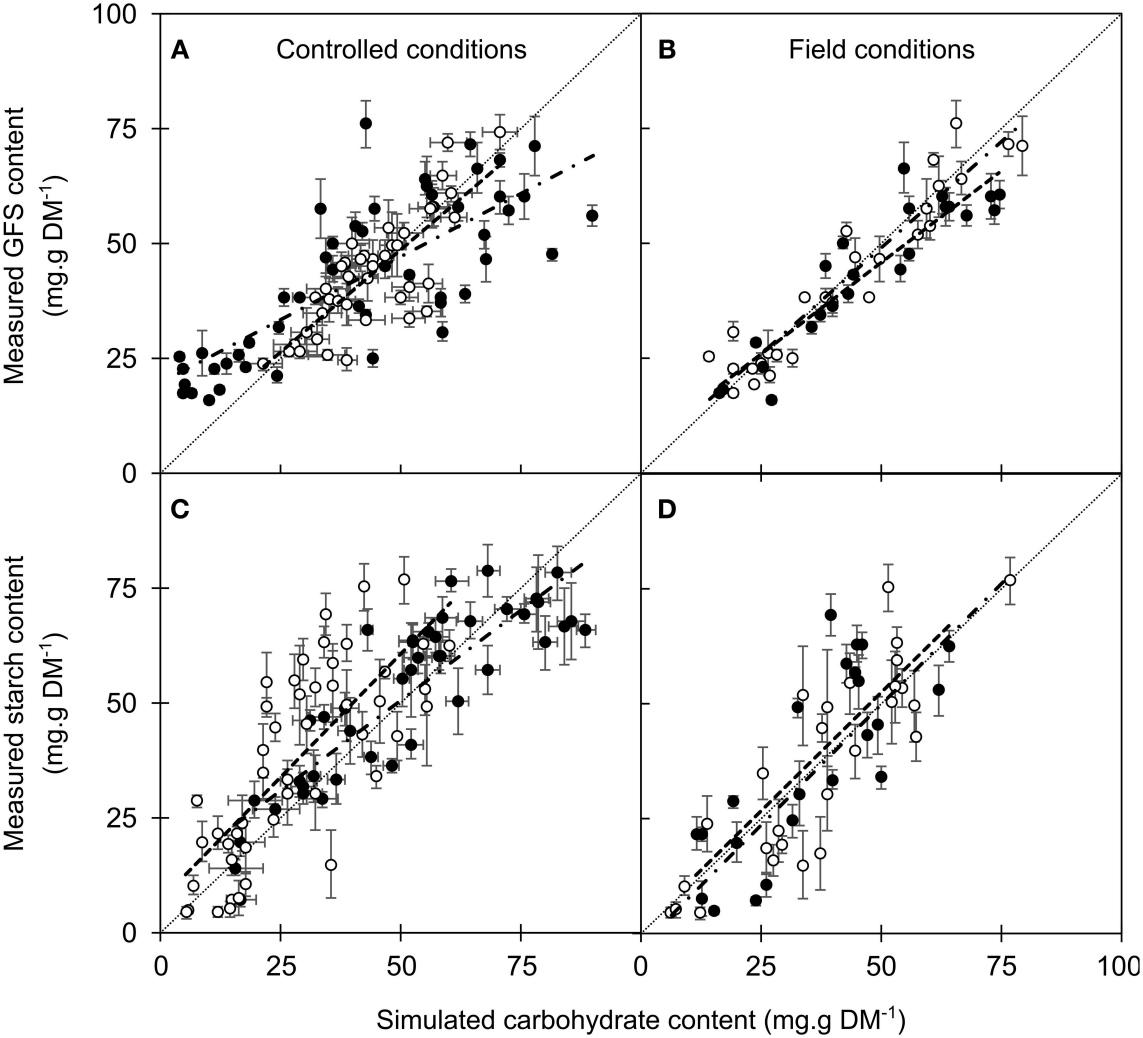
Measured vs. simulated values of soluble carbohydrates (GFS; **A,B**) and starch content **(C,D)**, respectively. The intermediate model was calibrated either on controlled **(A,C)** or field conditions **(B,D)**. Symbols and bars represent the mean and the standard error from 5–10 replicates (controlled conditions) or 5 replicates (field data) and open and closed circles represent calibration and validation datasets, respectively. Dashed and dot-and-dashed lines represent the linear regression between measured and simulated values in the calibration and validation datasets, respectively.

The different enzymatic reactions driving carbohydrate metabolism under the dependence of temperature, phenological stage and water content were modeled in accordance with these observation, including the allowed range for the different parameters (μ_i_ and σ_i_; Figures 2 –4; Table S3). The different models predicted the changes in GFS, with fairly good accuracy (RMSE = 11.12–11.78 g.g DM^−1^ and Eff = 0.355–0.426 for the different models; Table 2). The changes in starch content were more accurately predicted by the intermediate model (RMSE = 15.57 g.g DM−1; Eff = 0.530) than by the others (RMSE = 18.47 and 18.44, Eff = 0.340 and 0.341 for simple and complete version, respectively). For the intermediate model, the predicted values from NC dataset (i.e., field data) were relatively accurate for GFS (RMSEP = 15.19 mg.g DM^−1^ and Eff = 0.205) and for starch (16.61 mg.g DM^−1^ and Eff = 0.366; Figure 5). The two other models did not predict realistic values, with negative efficiencies and low accuracy (RMSEP > 30 mg.g DM−1 for GFS and Starch).

**Table 2:** Comparison between different models of carbohydrate metabolism depending on the architecture of the model (simple, intermediate, complete) and on the dataset used for calibration (CC and NC_1_ datasets).

**Dataset used for calibration**	**Model**	**Parameters**	***df***	**Calibration**				**Validation**			
				**GFS**		**Starch**		**GFS**		**Starch**	
				**RMSE (mg.g DM^−1^)**	**Eff**	**RMSE (mg.g DM^−1^)**	**Eff**	**RMSEP (mg.g DM^−1^)**	**Eff**	**RMSEP (mg.g DM^−1^)**	**Eff**
Controlled conditions	Simple	19	363	11.12	0.426	18.47	0.340	39.16	−0.474	31.82	−1.39
	Intermediate	19	363	11.78	0.355	15.57	0.530	15.19	0.205	16.61	0.366
	Complete	22	360	11.24	0.413	18.44	0.341	32.32	−0.004	41.98	−3.159
Natural conditions	Simple	19	116	9.78	0.896	15.44	0.460	15.04	0.831	25.15	−0.571
	Intermediate	19	116	5.86	0.962	10.56	0.752	7.84	0.951	12.65	0.626
	Complete	22	113	21.26	0.301	28.94	−1.259	17.71	0.765	19.71	0.035

**Figure 6:**
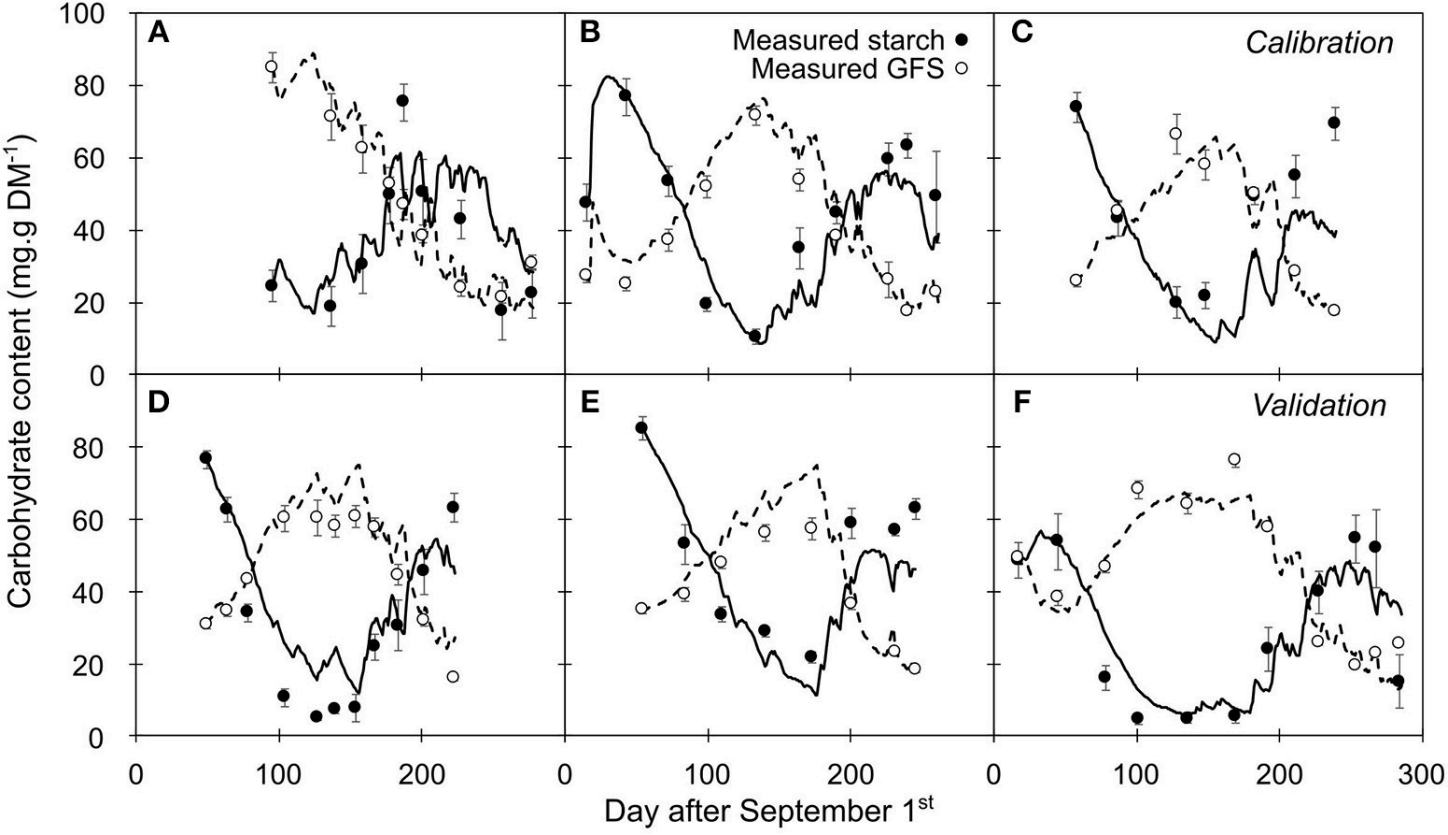
Seasonal variation in carbohydrate as measured and simulated by the intermediate model (GFS: open circle and dashed line, respectively; starch: closed circle and solid line, respectively). **(A–C**) Time series representing the data used for calibration. **(D–F)** External data used as a validation. Symbols and bars represent the mean and the standard error from five replicates.

The performance of the models to predict NC observations was improved when NC_1_ dataset was used for calibration. The simple and complete models both failed to properly simulate carbohydrates changes with NC_2_ dataset (RMSEP higher than 15, low Eff). However, the intermediate model was more accurate and efficient for both calibration (RMSE = 5.86 and 10.56 mg.g DM^−1^; Eff = 0.962 and 0.752 for GFS and Starch, respectively) and validation (RMSEP = 7.84 and 12.65 mg.g DM^−1^; Eff = 0.951 and 0.626 for GFS and Starch, respectively; Figures 5, 6). The selected years for calibration also affected the accuracy of the model to predict GFS and starch contents (Table S9). The highest accuracy was observed when the temperatures during autumn were the coldest and among the most variable (2007–2008 and 2008–2009).

The changes in GFS and starch contents were relatively well predicted for the calibration (NC_1_; Figures 6A–C) and validation dynamics (NC_2_; Figures 6D–F). However, although the accuracy in GFS prediction remained similar along the leafless period (RMSEP = 7.92 and 7.79 g.g−1 DM, for endodormancy and ecodormancy stage, respectively), starch content was more accurately predicted during endo (RMSEP = 9.58 g.g−1 DM) than during ecodormancy (RMSEP = 14.28 g.g−1 DM). This trend was more pronounced at the end of the endodormancy stage (RMSEP = 18.78 g.g−1 DM, when PS > 1.5) whereas GFS was still accurately predicted (RMSEP = 5.55 g.g−1 DM).

**Figure 7:**
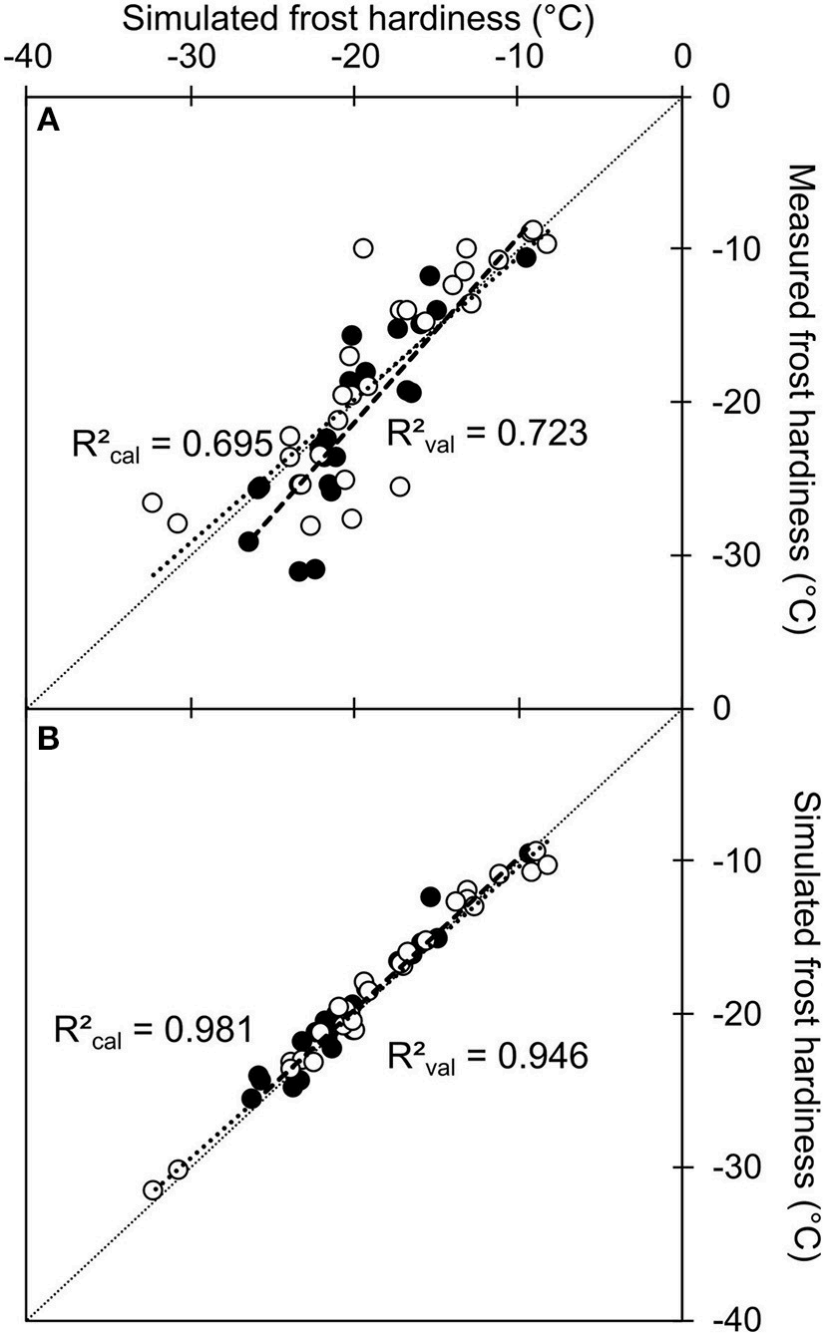
Simulated values of frost hardiness using the direct osmo-hydric model combined with the intermediate model simulating carbohydrate metabolism vs. measured values **(A)** or simulated values using the direct osmo hydric model alone **(B)**. The model used to calculate frost hardiness ($FH=a\cdot \frac{Ln\left(GFS\right)}{WC}+b$; Charrier et al., 2013a) was calibrated on measured GFS and WC value: *a* = −5.32 and *b* = 1.71. The calibration (open circles) and validation data (closed circles) were the same as Figure 6. Symbols represent the mean from five replicates (measured values). In both panels, short and long dashed lines represent the linear regression and respective regression coefficients (R^2^_cal_*and*R^2^_val_) between values in the calibration and validation datasets, respectively.

Finally, the accuracy and robustness of this model to predict frost hardiness was tested using the osmo-hydric model (depending on the relation between GFS and WC), calibrated on measured GFS and WC on calibration dataset. The prediction was very accurate with any of the tested dataset, and even slightly better using both models in combination: simulated *vs*. measured GFS values as input data, calibration vs. validation dataset (RMSE = 3.47°C and RMSEP = 3.55°C; RMSE = 3.68°C and RMSEP = 3.38°C, for the osmo-hydric model alone and combined with the intermediate model, respectively; Figure 7A). The discrepancy between both model was therefore very low (RMSE = 1.14°C), with no significant bias (*P* = 0.085; Figure 7B).

## Discussion

Carbon metabolism is a key component of plant ability to cope with environmental constraints (Hartmann and Trumbore, 2016), especially freezing stress during the dormant period (Gusta et al., 2004; Morin et al., 2007). Temperature affects the conversion between starch and soluble carbohydrates in different species e.g., *Populus sp, Salix sp* (Sakai, 1966c; Sauter, 1988). However, despite these primary observations, we were missing a model that could simulate the dynamics of carbohydrate composition, and related frost hardiness. Based on steady temperature treatments over 1–3 weeks, we tested different models to predict the dynamic equilibrium between these carbon pools. The most empirical and the most mechanistic models both failed to simulate properly the dynamics in GFS and starch under field conditions. However, the intermediate version (i.e., simulating the change in catalytic activity in relation with the substrate concentration, but without specified affinity) did accurately predict changes in GFS and starch over several years. The accuracy of the model was improved when the calibration was performed on field observations (NC_1_ dataset) rather from the controlled-conditions experiment (CC dataset). Furthermore, the selected years for calibration also affected the accuracy of the model to predict GFS and starch contents (Table S9). The highest accuracy was indeed observed when the temperatures during autumn were cold and variable. This observation confirms the importance of the temperature in the simulated process.

Different physiological variables, namely water and GFS contents, are relevant to predict frost hardiness in walnut trees (Poirier et al., 2010; Charrier et al., 2013a). Unfortunately, the measurement of these variables is time consuming and based on destructive analysis, which makes them only usable with difficulty for real-time predictions. However, the dynamic change in carbohydrate is driven by the temperature and the period of measurement (Sauter et al., 1996; Améglio et al., 2004; Poirier et al., 2010). Temperature is correlated with these physiological variables with an integrated response over 14–30 days (Figure 1; Table S1). It is interesting to note that although starch and GFS were better correlated over 30 and 14 days, respectively, frost hardiness which is correlated to both variables is better correlated over an intermediate time period (22 days). However, when data obtained at the edges of the leafless period (i.e., before leaf fall, or after budburst, i.e., sampled in mid-September or after May 10th, respectively) were included, the correlation was weaker (Figure 1), probably because carbon input (photosynthesis and leaf starch remobilization before leaf senescence) and outputs (root exportation and growth) occurs.

Nevertheless, the different correlations revealed an integrated response that could be explained by two factors: (i) the modulation of catalytic rate for the different reactions driving carbon metabolism (Witt and Sauter, 1994, 1995) and (ii) thermal inertia that could induce a deviation between air and organ temperature (Yamada and Takahashi, 2004). To minimize the effect of thermal inertia and previous thermal experience of the tree, sampled branches were kept under constant temperature for up to 3 weeks.

Temperature influences the carbohydrate metabolic rates as shown by the changes in NSC, GFS and starch contents over 1 and 3 weeks (Figure 2; Figures S1–S3). Interestingly, respiration, estimated by the change in NSCs was affected by both the temperature and its interaction with water content of the organ (Figure 2; Table S4). To date, the respiration of woody organs during the dormant period has been related to the carbohydrate content of the organ (Ögren, 2000). Here, we show that hydrated tissues would maintain higher metabolic activity, in interaction with temperature, accelerating the energy consumption over a period with low carbon input. Higher water contents during the growing season would increase the branch maintenance respiration compared to the leafless period, although Q_10_ remains relatively constant (*ca*. 1.7; Damesin et al., 2002). The value of the Q_10_ optimized by the model (Q_10_ = 2.64) was higher, although compatible with previous observation (e.g., 2.4 in *Quercus alba*, during the dormant period; Damesin et al., 2002). Finally, the interaction between warm temperature and high water content could therefore prevent trees from developing proper frost hardiness during autumn as shown by Charrier and Améglio (2011).

The controlled-temperature experiments revealed the influence of four factors on starch-to-soluble carbohydrate interconversion, namely temperature, season, years and treatment. Under controlled conditions, the normalization of the potential product by its respective substrate (GFS and starch by starch and GFS, respectively) decreased the heterogeneity across years and treatments (as revealed by the decrease in both variation coefficient and *P*-values; Tables S4–S6). Low temperature (<5°C) promoted starch hydrolysis and GFS increase across seasons, whereas, significant GFS increase was observed at warm temperature (>12°C) during autumn only (Figures 3–5). Although different enzymes (α-amylase, β-amylase, sucrose synthase, sucrose phosphate synthase) are involved in carbon metabolism during the dormant period (Witt and Sauter, 1994; Schrader and Sauter, 2002), their activity is modulated throughout the dormant period, as revealed by the change in GFS (Figure 5). The α-amylase activity is increasing at the onset of endodormancy (Elle and Sauter, 2000), and further progressively decreases (Witt and Sauter, 1994, 1995), as simulated by the model (Figures S4,S5). Another amylase activity could also significantly hydrolyze starch molecules. The β-amylase activity, which is induced by both cold and warm temperatures, would also be a good candidate (Kaplan and Guy, 2004). This enzyme has been observed in chloroplasts, vacuole and cytoplasm (Kaplan et al., 2006).

Integrating the results from controlled conditions experiment, three approaches were tested to simulate the catalytic rates driving carbon metabolism: an empirical (Simple: strict temperature control), an intermediate (temperature and substrate concentration control) and a mechanistic approach (complete: temperature, substrate concentration, and enzymatic affinity for the substrate). The respiration rate was modeled in relation to temperature and water content (Equation 6; Figure 2). The rate of starch to GFS conversion was highly variable across seasons (Figures 3–5). The enzymatic rates driving the reactions were modeled in relation to phenological stage (i.e., as a ratio of endo- and ecodormancy fulfillment: Equation 4). Although the three approaches were efficient to simulate the data used for calibration, only the intermediate approach was robust enough to simulate external values (Table 2). The empirical approach did not reflect biological reality (i.e., activity is limited by the presence of the substrate), whereas the mechanistic approach could not properly simulate the different reactions involved in the metabolic pathway (i.e., several enzymes with probably very different affinities k_M_). The intermediate model was the most accurate presumably because it reflects the underlying (enzymatic) processes, simulating the limiting chemical reaction, without integrating the possible variation in k_M_.

Finally, the model developed here was relatively efficient to predict change in starch, GFS and frost hardiness over several years under field conditions. This model included more simulated processes and related parameters than a straightforward model (e.g., osmo-hydric model). However, with respect to frost hardiness, the accuracy was similar to the osmo-hydric model previously developed on the same species (Charrier et al., 2013a; Figure 7), despite the propagation of uncertainty that is inherent to model coupling.

The prediction in starch content during the late ecodormancy (PS > 1.5) was less accurate whereas not in GFS. During this period, root water uptake is resumed when soil temperature reach 8°C, which induces the model to simulate higher losses in carbohydrate through respiration. Accurate measurements in respiration in relation with other physiological parameters would thus help to confirm this hypothesis (as stated Equation 6) as it may have major consequences on global carbon cycle in temperate areas, if warmer winters have to be expected. Warm temperatures and high water contents may lead to dramatic negative carbon balance that would affect both winter frost hardiness and spring growth resumption. However, despite the lower accuracy in starch content during spring, the prediction in frost hardiness remained fairly good for two reasons: (i) high water contents at this period has stronger influence on FH computation and (ii) GFS prediction remained good.

Even though more parameters have to be optimized than the osmo-hydric model, the model developed in this study provides an operative tool to simulate carbohydrate dynamics and related frost hardiness for the whole leafless period. It only requires one sampling date for initial carbohydrate content measurement at the beginning of endodormancy, and, relatively simple, water content and temperature data, avoiding costly and time-consuming measurements. In walnut trees, significant genotypic effect has been observed for eco-dormancy release and cold deacclimation (Charrier et al., 2011). Calibrating such a model on different walnut genotypes would bring new perspectives in identifying the variability in carbon metabolism related iso-enzymes and unraveling the genetic basis in frost hardiness variability (Guàrdia et al., 2016; Charrier et al., 2018). This could help the breeder for genetic analysis, especially when current production areas are expected to shift due to climate change. Other potential application in predicting the effect of warmer winters on dormancy and carbon metabolism (Pagter et al., 2015) and bud growing ability and organ development via the release of trophic limitation (Bonhomme et al., 2009). However, in its current state, the use of the current model for unraveling the genetic bases of FH variability or bud growth ability remains very speculative, and it should now deserve calibrating and testing under various meteorological conditions and biological systems.

## Author Contributions

GC and TA conceived the original screening and research plans; GC performed the experiments and measurements. GC and AL analyzed the data and developed the different models. GC wrote the article with contributions of all the authors.

### Conflict of Interest Statement

The authors declare that the research was conducted in the absence of any commercial or financial relationships that could be construed as a potential conflict of interest.
