# Supplementary material for: Dynamic Modeling of Carbon Metabolism During the Dormant Period Accurately Predicts the Changes in Frost Hardiness in Walnut Trees Juglans regia L
Source: Front Plant Sci. 2018 Dec 5;9:1746. doi: 10.3389/fpls.2018.01746 (PMC6290248; doi:10.3389/fpls.2018.01746)
Supplement: Supplementary file 6 [file Table_1.docx]

Supplementary material

Figure captions

**Figure S1.** Carbohydrate content measured during a controlled-temperature experiment at the beginning of endodormancy stage (late October – early November). Upper row: GFS (A) and starch (B) content after 7 (open symbols) and 20 days (closed symbols) storage at controlled temperature, depending on the temperature. Lower row: Change in GFS (C) and starch (D) content per day after 7 (open symbols) and 20 days (closed symbols) storage at controlled temperature, depending on the temperature. Symbol and bars represent the mean and Standard Error from n = 5 (Year 1 and 2) and 3 (Defoliated) replicates.

**Figure S2.** Carbohydrate content measured during a controlled-temperature experiment at the transition between endo- and ecodormancy stage (late January). Upper row: GFS (A) and starch (B) content after 7 (open symbols) and 20 days (closed symbols) storage at controlled temperature, depending on the temperature. Lower row: Change in GFS (C) and starch (D) content per day after 7 (open symbols) and 20 days (closed symbols) storage at controlled temperature, depending on the temperature. Symbol and bars represent the mean and Standard Error from n = 5 (Year 1 and 2) and 3 (Defoliated) replicates.

**Figure S3.** Carbohydrate content measured during a controlled-temperature experiment in the middle of the ecodormancy stage (late March). Upper row: GFS (A) and starch (B) content after 7 (open symbols) and 20 days (closed symbols) storage at controlled temperature, depending on the temperature. Lower row: Change in GFS (C) and starch (D) content per day after 7 (open symbols) and 20 days (closed symbols) storage at controlled temperature, depending on the temperature. Symbol and bars represent the mean and Standard Error from n = 5 (Year 1 and 2) and 3 (Defoliated) replicates.

**Figure S4. A.** Maximum catalytic rates (k_1c_, k_1m_ and k_2_) depending on phenological stage according to the calibration performed on controlled conditions dataset. **B-D**. Catalytic rates (k_1c_, k_1m_ and k_2_) depending on temperature at different phenological stages: onset of endodormancy (PS = 0; B); transition between endo-and ecodormancy (PS = 1; C) and budburst (PS = 2; D).

**Figure S5. A.** Maximum catalytic rates (k_1c_, k_1m_ and k_2_) depending on phenological stage according to the calibration performed on natural conditions dataset. **B-D**. Catalytic rates (k_1c_, k_1m_ and k_2_) depending on temperature at different phenological stages: onset of endodormancy (PS = 0; B); transition between endo-and ecodormancy (PS = 1; C) and budburst (PS = 2; D).

**Table S1.** Pearson product-moment correlation coefficients between frost hardiness, water content, soluble carbohydrate content, or starch content, and mean temperature of either daily maximal, daily minimal or daily average temperatures calculated from 0 (sampling date) to 30 days before sampling. All correlations were significant (P < 0.0001, except for water content and mean temperature calculated for less than 3 days, where P < 0.0005). The most significant correlation is indicated in bold. These calculations reveal the delay for physiological modulations to take place in response to variations in environmental conditions.

| Factor | Frost hardiness | Water content | Soluble Carbohydrate | Starch | Starch  (leafless period) |
| --- | --- | --- | --- | --- | --- |
| Temperature | Maximum | Minimum | Maximum | Maximum | Maximum |
| Day 0  (sampling date) | 0.7879 | 0.5199 | -0.6825 | 0.5437 | 0.6521 |
| Day 0 to -1 | 0.8183 | 0.5196 | -0.7220 | 0.5696 | 0.6894 |
| Day 0 to -2 | 0.8153 | 0.5334 | -0.7342 | 0.5861 | 0.7124 |
| Day 0 to -3 | 0.8214 | 0.5502 | -0.7393 | 0.6024 | 0.7384 |
| Day 0 to -4 | 0.8220 | 0.5605 | -0.7459 | 0.6084 | 0.7419 |
| Day 0 to -5 | 0.8235 | 0.5627 | -0.7641 | 0.6121 | 0.7478 |
| Day 0 to -6 | 0.8370 | 0.5900 | -0.7798 | 0.6177 | 0.7637 |
| Day 0 to -7 | 0.8495 | 0.6021 | -0.7861 | 0.6348 | 0.7917 |
| Day 0 to -8 | 0.8564 | 0.6245 | -0.7875 | 0.6448 | 0.8059 |
| Day 0 to -9 | 0.8580 | 0.6276 | -0.7945 | 0.6493 | 0.8123 |
| Day 0 to -10 | 0.8612 | 0.6278 | -0.7952 | 0.6522 | 0.8237 |
| Day 0 to -11 | 0.8665 | 0.6226 | -0.7969 | 0.6573 | 0.8348 |
| Day 0 to -12 | 0.8734 | 0.6124 | -0.7951 | 0.6639 | 0.8444 |
| Day 0 to -13 | 0.8768 | 0.6202 | -0.7965 | 0.6713 | 0.8520 |
| Day 0 to -14 | 0.8821 | 0.6347 | **-0.7999** | 0.6793 | 0.8591 |
| Day 0 to -15 | 0.8849 | 0.6385 | -0.7951 | **0.6807** | 0.8623 |
| Day 0 to -16 | 0.8850 | 0.6452 | -0.7910 | 0.6730 | 0.8610 |
| Day 0 to -17 | 0.8905 | 0.6551 | -0.7912 | 0.6674 | 0.8633 |
| Day 0 to -18 | 0.8953 | 0.6571 | -0.7890 | 0.6612 | 0.8605 |
| Day 0 to -19 | 0.8954 | 0.6625 | -0.7878 | 0.6590 | 0.8608 |
| Day 0 to -20 | 0.8950 | 0.6674 | -0.7868 | 0.6589 | 0.8625 |
| Day 0 to -21 | 0.8954 | 0.6715 | -0.7840 | 0.6606 | 0.8674 |
| Day 0 to -22 | **0.8955** | 0.6704 | -0.7828 | 0.6632 | 0.8716 |
| Day 0 to -23 | 0.8951 | **0.6737** | -0.7821 | 0.6668 | 0.8763 |
| Day 0 to -24 | 0.8944 | 0.6713 | -0.7794 | 0.6689 | 0.8790 |
| Day 0 to -25 | 0.8899 | 0.6678 | -0.7740 | 0.6687 | 0.8789 |
| Day 0 to -26 | 0.8869 | 0.6634 | -0.7706 | 0.6705 | 0.8803 |
| Day 0 to -27 | 0.8850 | 0.6648 | -0.7704 | 0.6760 | 0.8832 |
| Day 0 to -28 | 0.8805 | 0.6652 | -0.7690 | 0.6782 | 0.8835 |
| Day 0 to -29 | 0.8764 | 0.6649 | -0.7667 | 0.6782 | 0.8826 |
| Day 0 to -30 | 0.8745 | 0.6622 | -0.7638 | 0.6764 | **0.8836** |

**Table S2.** Description of the data used for calibration and validation of the model (C and V, respectively), including the location and date of sampling, the temperature treatment and number of replicates.

| Dataset | CC Calibration | NC Calibration | Year | Location | Date | Temperature  treatment | n |
| --- | --- | --- | --- | --- | --- | --- | --- |
| NC_1_ | V | C | 1 | Lowland | 12/05/07 | Field | 5 |
| NC_1_ | V | C | 1 | Lowland | 01/15/08 | Field | 5 |
| NC_1_ | V | C | 1 | Lowland | 02/06/08 | Field | 5 |
| NC_1_ | V | C | 1 | Lowland | 02/25/08 | Field | 5 |
| NC_1_ | V | C | 1 | Lowland | 03/05/08 | Field | 5 |
| NC_1_ | V | C | 1 | Lowland | 03/19/08 | Field | 5 |
| NC_1_ | V | C | 1 | Lowland | 04/14/08 | Field | 5 |
| NC_1_ | V | C | 1 | Lowland | 05/13/08 | Field | 5 |
| NC_1_ | V | C | 1 | Lowland | 06/03/08 | Field | 5 |
| NC_1_ | V | C | 2 | Lowland | 09/15/08 | Field | 5 |
| NC_1_ | V | C | 2 | Lowland | 10/13/08 | Field | 5 |
| NC_1_ | V | C | 2 | Lowland | 11/12/08 | Field | 5 |
| NC_1_ | V | C | 2 | Lowland | 12/08/08 | Field | 5 |
| NC_1_ | V | C | 2 | Lowland | 01/12/09 | Field | 5 |
| NC_1_ | V | C | 2 | Lowland | 02/11/09 | Field | 5 |
| NC_1_ | V | C | 2 | Lowland | 03/09/09 | Field | 5 |
| NC_1_ | V | C | 2 | Lowland | 04/14/09 | Field | 5 |
| NC_1_ | V | C | 2 | Lowland | 04/28/09 | Field | 5 |
| NC_1_ | V | C | 2 | Lowland | 05/18/09 | Field | 5 |
| NC_1_ | V | C | 3 | Mountain | 10/15/08 | Field | 5 |
| NC_1_ | V | C | 3 | Mountain | 11/17/08 | Field | 5 |
| NC_1_ | V | C | 3 | Mountain | 12/10/08 | Field | 5 |
| NC_1_ | V | C | 3 | Mountain | 01/14/09 | Field | 5 |
| NC_1_ | V | C | 3 | Mountain | 02/16/09 | Field | 5 |
| NC_1_ | V | C | 3 | Mountain | 03/11/09 | Field | 5 |
| NC_1_ | V | C | 3 | Mountain | 04/15/09 | Field | 5 |
| NC_1_ | V | C | 3 | Mountain | 05/11/09 | Field | 5 |
| NC_1_ | V | C | 3 | Mountain | 05/25/09 | Field | 5 |
| NC_1_ | V | C | 3 | Mountain | 06/11/09 | Field | 5 |
| NC_2_ | V | V | 4 | Lowland | 10/28/09 | Field | 5 |
| NC_2_ | V | V | 4 | Lowland | 11/26/09 | Field | 5 |
| NC_2_ | V | V | 4 | Lowland | 01/06/10 | Field | 5 |
| NC_2_ | V | V | 4 | Lowland | 01/26/10 | Field | 5 |
| NC_2_ | V | V | 4 | Lowland | 03/01/10 | Field | 5 |
| NC_2_ | V | V | 4 | Lowland | 03/30/10 | Field | 5 |
| NC_2_ | V | V | 4 | Lowland | 04/27/10 | Field | 5 |
| NC_2_ | V | V | 5 | Lowland | 10/20/10 | Field | 5 |
| NC_2_ | V | V | 5 | Lowland | 11/02/10 | Field | 5 |
| NC_2_ | V | V | 5 | Lowland | 11/17/10 | Field | 5 |
| NC_2_ | V | V | 5 | Lowland | 12/13/10 | Field | 5 |
| NC_2_ | V | V | 5 | Lowland | 01/05/11 | Field | 5 |
| NC_2_ | V | V | 5 | Lowland | 01/17/11 | Field | 5 |
| NC_2_ | V | V | 5 | Lowland | 02/02/11 | Field | 5 |
| NC_2_ | V | V | 5 | Lowland | 02/14/11 | Field | 5 |
| NC_2_ | V | V | 5 | Lowland | 03/02/11 | Field | 5 |
| NC_2_ | V | V | 5 | Lowland | 03/21/11 | Field | 5 |
| NC_2_ | V | V | 5 | Lowland | 04/11/11 | Field | 5 |
| NC_2_ | V | V | 6 | Mountain | 10/24/11 | Field | 5 |
| NC_2_ | V | V | 6 | Mountain | 11/23/11 | Field | 5 |
| NC_2_ | V | V | 6 | Mountain | 12/19/11 | Field | 5 |
| NC_2_ | V | V | 6 | Mountain | 01/18/12 | Field | 5 |
| NC_2_ | V | V | 6 | Mountain | 02/20/12 | Field | 5 |
| NC_2_ | V | V | 6 | Mountain | 03/19/12 | Field | 5 |
| NC_2_ | V | V | 6 | Mountain | 04/18/12 | Field | 5 |
| NC_2_ | V | V | 6 | Mountain | 05/02/12 | Field | 5 |
| NC_2_ | V | V | 6 | Mountain | 09/17/08 | Field | 5 |
| CC (Year 1) | C | *NA* |  | Lowland | 10/29/08 | -3 | 5 |
| CC (Year 1) | C | *NA* |  | Lowland | 10/29/08 | 1 | 5 |
| CC (Year 1) | C | *NA* |  | Lowland | 10/29/08 | 5 | 5 |
| CC (Year 1) | C | *NA* |  | Lowland | 10/29/08 | 10 | 5 |
| CC (Year 1) | C | *NA* |  | Lowland | 10/29/08 | 15 | 5 |
| CC (Year 1) | C | *NA* |  | Lowland | 10/29/08 | 20 | 5 |
| CC (Year 1) | C | *NA* |  | Lowland | 10/29/08 | 25 | 5 |
| CC (Year 1) | C | *NA* |  | Lowland | 11/10/08 | -3 | 5 |
| CC (Year 1) | C | *NA* |  | Lowland | 11/10/08 | 1 | 5 |
| CC (Year 1) | C | *NA* |  | Lowland | 11/10/08 | 5 | 5 |
| CC (Year 1) | C | *NA* |  | Lowland | 11/10/08 | 10 | 5 |
| CC (Year 1) | C | *NA* |  | Lowland | 11/10/08 | 15 | 5 |
| CC (Year 1) | C | *NA* |  | Lowland | 11/10/08 | 20 | 5 |
| CC (Year 1) | C | *NA* |  | Lowland | 11/10/08 | 25 | 5 |
| CC (Year 1) | C | *NA* |  | Lowland | 01/26/09 | -3 | 5 |
| CC (Year 1) | C | *NA* |  | Lowland | 01/26/09 | 1 | 5 |
| CC (Year 1) | C | *NA* |  | Lowland | 01/26/09 | 5 | 5 |
| CC (Year 1) | C | *NA* |  | Lowland | 01/26/09 | 10 | 5 |
| CC (Year 1) | C | *NA* |  | Lowland | 01/26/09 | 15 | 5 |
| CC (Year 1) | C | *NA* |  | Lowland | 01/26/09 | 20 | 5 |
| CC (Year 1) | C | *NA* |  | Lowland | 01/26/09 | 25 | 5 |
| CC (Year 1) | C | *NA* |  | Lowland | 02/09/09 | -3 | 5 |
| CC (Year 1) | C | *NA* |  | Lowland | 02/09/09 | 1 | 5 |
| CC (Year 1) | C | *NA* |  | Lowland | 02/09/09 | 5 | 5 |
| CC (Year 1) | C | *NA* |  | Lowland | 02/09/09 | 10 | 5 |
| CC (Year 1) | C | *NA* |  | Lowland | 02/09/09 | 15 | 5 |
| CC (Year 1) | C | *NA* |  | Lowland | 02/09/09 | 20 | 5 |
| CC (Year 1) | C | *NA* |  | Lowland | 02/09/09 | 25 | 5 |
| CC (Year 1) | C | *NA* |  | Lowland | 03/24/09 | -3 | 5 |
| CC (Year 1) | C | *NA* |  | Lowland | 03/24/09 | 1 | 5 |
| CC (Year 1) | C | *NA* |  | Lowland | 03/24/09 | 5 | 5 |
| CC (Year 1) | C | *NA* |  | Lowland | 03/24/09 | 10 | 5 |
| CC (Year 1) | C | *NA* |  | Lowland | 03/24/09 | 15 | 5 |
| CC (Year 1) | C | *NA* |  | Lowland | 03/24/09 | 20 | 5 |
| CC (Year 1) | C | *NA* |  | Lowland | 03/24/09 | 25 | 5 |
| CC (Year 1) | C | *NA* |  | Lowland | 04/07/09 | -3 | 5 |
| CC (Year 1) | C | *NA* |  | Lowland | 04/07/09 | 1 | 5 |
| CC (Year 1) | C | *NA* |  | Lowland | 04/07/09 | 5 | 5 |
| CC (Year 1) | C | *NA* |  | Lowland | 04/07/09 | 10 | 5 |
| CC (Year 1) | C | *NA* |  | Lowland | 04/07/09 | 15 | 5 |
| CC (Year 1) | C | *NA* |  | Lowland | 04/07/09 | 20 | 5 |
| CC (Year 1) | C | *NA* |  | Lowland | 04/07/09 | 25 | 5 |
| CC (Year 2) | C | *NA* |  | Lowland | 11/04/09 | -3 | 8 (5 control + 3 defoliated) |
| CC (Year 2) | C | *NA* |  | Lowland | 11/04/09 | 1 | 5 control |
| CC (Year 2) | C | *NA* |  | Lowland | 11/04/09 | 5 | 5 control |
| CC (Year 2) | C | *NA* |  | Lowland | 11/04/09 | 10 | 5 control |
| CC (Year 2) | C | *NA* |  | Lowland | 11/04/09 | 15 | 8 (5 control + 3 defoliated) |
| CC (Year 2) | C | *NA* |  | Lowland | 11/04/09 | 20 | 5 control |
| CC (Year 2) | C | *NA* |  | Lowland | 11/04/09 | 25 | 8 (5 control + 3 defoliated) |
| CC (Year 2) | C | *NA* |  | Lowland | 11/17/09 | -3 | 8 (5 control + 3 defoliated) |
| CC (Year 2) | C | *NA* |  | Lowland | 11/17/09 | 1 | 5 control |
| CC (Year 2) | C | *NA* |  | Lowland | 11/17/09 | 5 | 5 control |
| CC (Year 2) | C | *NA* |  | Lowland | 11/17/09 | 10 | 5 control |
| CC (Year 2) | C | *NA* |  | Lowland | 11/17/09 | 15 | 8 (5 control + 3 defoliated) |
| CC (Year 2) | C | *NA* |  | Lowland | 11/17/09 | 20 | 5 control |
| CC (Year 2) | C | *NA* |  | Lowland | 11/17/09 | 25 | 8 (5 control + 3 defoliated) |
| CC (Year 2) | C | *NA* |  | Lowland | 02/02/10 | -3 | 8 (5 control + 3 defoliated) |
| CC (Year 2) | C | *NA* |  | Lowland | 02/02/10 | 15 | 8 (5 control + 3 defoliated) |
| CC (Year 2) | C | *NA* |  | Lowland | 02/02/10 | 25 | 8 (5 control + 3 defoliated) |
| CC (Year 2) | C | *NA* |  | Lowland | 02/15/10 | -3 | 8 (5 control + 3 defoliated) |
| CC (Year 2) | C | *NA* |  | Lowland | 02/15/10 | 15 | 8 (5 control + 3 defoliated) |
| CC (Year 2) | C | *NA* |  | Lowland | 02/15/10 | 25 | 8 (5 control + 3 defoliated) |
| CC (Year 2) | C | *NA* |  | Lowland | 04/06/10 | -3 | 8 (5 control + 3 defoliated) |
| CC (Year 2) | C | *NA* |  | Lowland | 04/06/10 | 15 | 8 (5 control + 3 defoliated) |
| CC (Year 2) | C | *NA* |  | Lowland | 04/06/10 | 25 | 8 (5 control + 3 defoliated) |
| CC (Year 2) | C | *NA* |  | Lowland | 04/19/10 | -3 | 8 (5 control + 3 defoliated) |
| CC (Year 2) | C | *NA* |  | Lowland | 04/19/10 | 15 | 8 (5 control + 3 defoliated) |
| CC (Year 2) | C | *NA* |  | Lowland | 04/19/10 | 25 | 8 (5 control + 3 defoliated) |

**Table S3.** Optimized parameters for the different models calibrated from controlled-conditions experiment (CC) or natural conditions observations (NC).

| **Parameter** | **Description** | **Simple** | | **Intermediate** | | **Michaelis - Menten** | | **Unit** | **Allowed range** | | |
| --- | --- | --- | --- | --- | --- | --- | --- | --- | --- | --- | --- |
|  |  | **CC** | **NC** | **CC** | **NC** | **CC** | **NC** |  | | **Min** | **Max** |
| Cold hydrolysis (k_1c_) | |  |  |  |  |  |  |  | |  |  |
| µ_1c_ | Optimal temperature for enzymatic activity | 0.73 | 5.00 | -10.00 | 8.00 | 2.67 | 0.67 | °C | | -10 | 8 |
| σ_1c_ | Standard deviation for enzymatic activity | 25.00 | 6.39 | 22.68 | 7.69 | 25.00 | 21.36 | °C | | 2.5 | 25 |
| K_M1c_ | Michaelis constant | *NA* | *NA* | *NA* | *NA* | 0.286 | 0.507 | mg.g DM^-1^ | | 0 | 5 |
| k_1c trans_ | Catalytic rate at the transition between endo- and ecodormancy | 4.66 | -0.00351 | 0.0657 | 0.0997 | 0.835 | 2.01 | *Unitless* | | *free* | *free* |
| d_endo1c_ | Difference between a_1c endo_ and b_1c endo_ | 2.53 | 0.574 | 0.0523 | 0.0888 | -0.106 | 0.809 | *Unitless* | | *free* | *free* |
| d_eco1c_ | Difference between a_1c eco_ and b_1c eco_ | -0.327 | -2.09 | -0.164 | 0.0795 | -0.0376 | -0.425 | *Unitless* | | *free* | *free* |
| Mild starch hydrolysis (k_1m_) | |  |  |  |  |  |  |  | |  |  |
| µ_1m_ | Optimal temperature for enzymatic activity | 22.60 | 33.85 | 23.23 | 19.71 | 22.76 | 22.76 | °C | | 12.5 | 40 |
| σ_1m_ | Standard deviation for enzymatic activity | 2.50 | 2.50 | 6.59 | 2.59 | 2.50 | 2.50 | °C | | 2.5 | 25 |
| K_M1m_ | Michaelis constant | *NA* | *NA* | *NA* | *NA* | 0.483 | 0.511 | mg.g DM^-1^ | | 0 | 5 |
| k_1m trans_ | Catalytic rate at the transition between endo- and ecodormancy | -1.65 | 38.40 | -0.138 | -217.3 | -0.00704 | -0.75 | *Unitless* | | *free* | *free* |
| d_endo1m_ | Difference between a_1m endo_ and b_1m endo_ | -3.64 | -14.10 | -0.294 | -257.6 | -0.0180 | -1.65 | *Unitless* | | *free* | *free* |
| d_eco1m_ | Difference between a_1m eco_ and b_1m eco_ | 12.01 | 67.40 | 0.514 | 0.650 | 3.26 | 3.19 | *Unitless* | | *free* | *free* |
| Starch synthesis (k_2_) | |  |  |  |  |  |  |  | |  |  |
| µ_2_ | Optimal temperature for enzymatic activity | 12.50 | 12.50 | 12.09 | 10 | 22.24 | 12.50 | °C | | 10 | 40 |
| σ_2_ | Standard deviation for enzymatic activity | 23.51 | 6.01 | 11.57 | 3.77 | 25.00 | 22.08 | °C | | 2.5 | 25 |
| K_M2_ | Michaelis constant | *NA* | *NA* | *NA* | *NA* | 0.0862 | 0.260 | mg.g DM^-1^ | | 0 | 5 |
| k_2 trans_ | Catalytic rate at the transition between endo- and ecodormancy | 5.25 | 1.99 | 0.0296 | 0.0464 | 0.356 | 1.38 | *Unitless* | | *free* | *free* |
| d_endo 2_ | Difference between a_2 endo_ and b_2 endo_ | 8.90 | 27.56 | 1.709 | -0.0633 | 0.603 | -1.80 | *Unitless* | | *free* | *free* |
| d_eco2_ | Difference between a_2 eco_ and b_2 eco_ | -4.21 | -9.44 | 0.0645 | 0.856 | -0.398 | -1.36 | *Unitless* | | *free* | *free* |
| Respiration (k_3_) | |  |  |  |  |  |  |  | |  |  |
| Q_10_ | Q10 coefficient | 1.47 | 1.00 | 1.559 | 2.64 | 1.46 | 1.507 |  | | 1 | 5 |
| a_3_ | Sensitivity to water content | -3.15 | -0.00178 | -0.455 | -165.5 | -6.98 | 0.0222 |  | | *free* | *free* |
| b_3_ | WC50 | 1.06 | 0.80 | 0.800 | 0.924 | 0.800 | 1.05 |  | | 0.8 | 1.5 |
| R_Max_ | Maximum respiration rate | 1.00 | 0.291 | 1.00 | 0.693 | 0.566 | 0.689 |  | | *free* | *free* |

**Table S4.** Linear model between the variation in total dNSC during controlled experiments (log transformed), initial water content and temperature, including their interaction.

| Factors | Estimate | Standard Error | t-value | P-value |
| --- | --- | --- | --- | --- |
| Water Content | 0.3901 | 2.2826 | 0.171 | 0.8645 |
| Temperature | -0.3208 | 0.1402 | -2.288 | 0.0233 * |
| Temperature * Water Content | 0.3889 | 0.1559 | 2.494 | 0.0135 * |
| Intercept | -2.6283 | 2.0710 | -1.269 | 0.2060 |

**Table S5.** Probabilities of significant differences calculated by Kruskal-Wallis test (P-values) in the change in carbohydrates during the controlled temperature experiment (dGFS or dStarch, normalized or not by the potential substrate, Starch and GFS, respectively) across seasons (for the complete dataset or at different temperature), temperature (for the complete dataset or at different season) or their interaction according to the non-parametric Wilcoxson test (^ns^: P>0.05; * P<0.05; ** P< 0.01; ***; P < 0.001).

| **Principal effect** | **Data** | **dGFS / dt** | **dGFS / dt . Starch** | **dStarch / dt** | **dStarch / dt . GFS** |
| --- | --- | --- | --- | --- | --- |
| **Season** | **All** | <0.0001 *** | <0.0001 *** | <0.0001 *** | <0.0001 *** |
|  | **-3** | <0.0001 *** | 0.2646 ^ns^ | 0.0031 ** | 0.0001 *** |
|  | **1** | <0.0001 *** | 0.0002 *** | <0.0001 *** | <0.0001 *** |
|  | **5** | 0.0002 *** | 0.0008 *** | 0.0001 *** | 0.0002 *** |
|  | **10** | 0.0001 *** | <0.0001 *** | <0.0001 *** | 0.0002 *** |
|  | **15** | <0.0001 *** | <0.0001 *** | <0.0001 *** | <0.0001 *** |
|  | **20** | <0.0001 *** | <0.0001 *** | 0.0064 ** | 0.0231 * |
|  | **25** | <0.0001 *** | <0.0001 *** | <0.0001 *** | 0.0004 *** |
| **Temperature** | **All** | <0.0001 *** | <0.0001 *** | <0.0001 *** | 0.0001 *** |
|  | **Autumn** | <0.0001 *** | <0.0001 *** | 0.3499 ^ns^ | 0.3281 ^ns^ |
|  | **Winter** | <0.0001 *** | <0.0001 *** | <0.0001 *** | <0.0001 *** |
|  | **Spring** | <0.0001 *** | <0.0001 *** | 0.0076 ** | 0.0048 ** |
| **Season x Temperature** | **All** | <0.0001 *** | <0.0001 *** | <0.0001 *** | <0.0001 *** |
| **Year** | **All** | 0.0001 *** | <0.0001 *** | 0.0015 ** | 0.0024 ** |
| **Treatment** | **-3; 15; 25°C** | 0.0081 ** | 0.0570 ^ns^ | 0.8106 ^ns^ | 0.7440 ^ns^ |

**Table S6.** Probabilities of significant differences calculated by Kruskal-Wallis test (P-values) in carbohydrates (dGFS or dStarch, normalized or not by the potential substrate, Starch and GFS, respectively) between years (control trees: Year1 vs Year 2) according to the non-parametric Wilcoxson test (^ns^: P>0.05; * P<0.05; ** P< 0.01; ***; P < 0.001).

| **Season** | **Temperature** | **dGFS / dt** | **dGFS / dt . Starch** | **dStarch / dt** | **dStarch / dt . GFS** |
| --- | --- | --- | --- | --- | --- |
| **Autumn** | **-3** | 0.0288 * | 0.1230 ^ns^ | 0.0021 ** | 0.0288 * |
|  | **1** | 0.1321 ^ns^ | 0.8094 ^ns^ | 0.0011 ** | 0.0197 * |
|  | **5** | 0.0005 *** | 0.0005 *** | 0.0524 ^ns^ | 0.1051 ^ns^ |
|  | **10** | 0.0015 ** | 0.0006 *** | 0.0015 ** | 0.0021 ** |
|  | **15** | 0.1431 ^ns^ | 0.0355 * | 0.0011 ** | 0.0039 ** |
|  | **20** | 0.0041 ** | 0.0003 *** | 0.0030 ** | 0.0057 ** |
|  | **25** | 0.5135 ^ns^ | 0.8591 ^ns^ | 0.0007 *** | 0.0007 *** |
| **Winter** | **-3** | 0.0288 * | 0.0433 * | 1.0000 ^ns^ | 0.8534 ^ns^ |
|  | **15** | 0.1655 ^ns^ | 0.0753 ^ns^ | 0.0753 ^ns^ | 0.0753 ^ns^ |
|  | **25** | 0.1230 ^ns^ | 0.1431 ^ns^ | 0.2176 ^ns^ | 0.1230 ^ns^ |
| **Spring** | **-3** | 0.1903 ^ns^ | 0.1230 ^ns^ | 0.0115 * | 0.0433 * |
|  | **15** | 0.1431 ^ns^ | 0.4359 ^ns^ | 0.1903 ^ns^ | 0.1655 ^ns^ |
|  | **25** | 0.6305 ^ns^ | 0.7959 ^ns^ | 0.2176 ^ns^ | 0.2475 ^ns^ |

**Table S7.** Probabilities of significant differences (P-values) in carbohydrates (dGFS or dStarch, normalized or not by the potential substrate, Starch and GFS, respectively) between treatments (Year2: control vs defoliated) according to the non-parametric Wilcoxson test (^ns^: P>0.05; * P<0.05; ** P< 0.01; ***; P < 0.001).

| **Season** | **Temperature** | **dGFS / dt** | **dGFS / dt . Starch** | **dStarch / dt** | **dStarch / dt . GFS** |
| --- | --- | --- | --- | --- | --- |
| **Autumn** | **-3** | 0.9578 ^ns^ | 0.9578 ^ns^ | 0.1471 ^ns^ | 0.2198 ^ns^ |
|  | **15** | 0.2198 ^ns^ | 0.6354 ^ns^ | 0.2635 ^ns^ | 0.3132 ^ns^ |
|  | **25** | 0.0110 * | 0.0225 * | 0.9578 ^ns^ | 0.9578 ^ns^ |
| **Winter** | **-3** | 0.7128 ^ns^ | 0.4278 ^ns^ | 0.1179 ^ns^ | 0.4278 ^ns^ |
|  | **15** | 0.0075 ** | 0.0110 * | 0.3132 ^ns^ | 0.2635 ^ns^ |
|  | **25** | 0.0312 * | 0.0420 * | 0.2198 ^ns^ | 0.1179 ^ns^ |
| **Spring** | **-3** | 0.1806 ^ns^ | 0.1471 ^ns^ | 0.6354 ^ns^ | 0.7925 ^ns^ |
|  | **15** | 0.0559 ^ns^ | 0.0934 ^ns^ | 0.2198 ^ns^ | 0.6354 ^ns^ |
|  | **25** | 0.1179 ^ns^ | 0.1471 ^ns^ | 0.6354 ^ns^ | 0.9578 ^ns^ |

| **Season** | **Temperature** | **dGFS / dt** | **dGFS / dt . Starch** | **dStarch / dt** | **dStarch / dt . GFS** |
| --- | --- | --- | --- | --- | --- |
| **Autumn** | **-3** | 0.1292 ^ns^ | 0.4866 ^ns^ | 0.0075 ** | 0.0565 ^ns^ |
|  | **1** | 0.1321 ^ns^ | 0.8094 ^ns^ | 0.0011 ** | 0.0197 * |
|  | **5** | 0.0005 *** | 0.0005 *** | 0.0524 ^ns^ | 0.1051 ^ns^ |
|  | **10** | 0.0015 ** | 0.0006 *** | 0.0015 ** | 0.0021 ** |
|  | **15** | 0.2325 ^ns^ | 0.0778 ^ns^ | 0.0074 ** | 0.0193 * |
|  | **20** | 0.0041 ** | 0.0003 *** | 0.0030 ** | 0.0057 ** |
|  | **25** | 0.0266 * | 0.0651 ^ns^ | 0.0043 ** | 0.0056 ** |
| **Winter** | **-3** | 0.1021 ^ns^ | 0.1158 ^ns^ | 0.1889 ^ns^ | 0.3837 ^ns^ |
|  | **15** | 0.2327 ^ns^ | 0.0216* | 0.1907 ^ns^ | 0.1824 ^ns^ |
|  | **25** | 0.0673 ^ns^ | 0.0804 ^ns^ | 0.2929 ^ns^ | 0.1346 ^ns^ |
| **Spring** | **-3** | 0.2325 ^ns^ | 0.1728 ^ns^ | 0.0393 * | 0.1125 ^ns^ |
|  | **15** | 0.0983 ^ns^ | 0.1754 ^ns^ | 0.1353 ^ns^ | 0.2465 ^ns^ |
|  | **25** | 0.1914 ^ns^ | 0.1669 ^ns^ | 0.3422 ^ns^ | 0.3101 ^ns^ |

**Tab. S8.** Probabilities of significant differences (P-values) in carbohydrates (dGFS or dStarch, normalized or not by the potential substrate, Starch and GFS, respectively) between years and treatments (Year1 vs Year2 control vs Year2 defoliated) according to the non-parametric Wilcoxson test (^ns^: P>0.05; * P<0.05; ** P< 0.01; ***; P < 0.001).

**Tab S9.** Results (RMSE and RMSEP) from independent calibration depending on the selected winter dynamics for calibration (3 out of 6). The average temperature and standard deviation during autumn, winter spring and the three season for the selected years are indicated.

| Selected years for calibration | | | Starch & GFS | | GFS | | Starch | | Temperature (mean ± SD) | | | |
| --- | --- | --- | --- | --- | --- | --- | --- | --- | --- | --- | --- | --- |
|  |  |  | RMSE | RMSEP | RMSE | RMSEP | RMSE | RMSEP | Autumn | Winter | Spring | All period |
| 1 | 2 | 3 | 8.80 | 10.81 | 5.08 | 9.83 | 11.37 | 11.71 | 11.77 ± 5.17 | 3.83 ± 4.38 | 11.04 ± 4.87 | 8.9 ± 6 |
| 1 | 2 | 4 | 7.79 | 14.41 | 5.94 | 12.04 | 9.28 | 16.43 | 11.17 ± 5.35 | 3.98 ± 4.31 | 11.93 ± 4.72 | 9.05 ± 5.99 |
| 1 | 2 | 5 | 8.51 | 11.16 | 5.86 | 8.28 | 10.52 | 13.45 | 12.11 ± 5.31 | 4.03 ± 4.71 | 11.53 ± 4.56 | 9.24 ± 6.1 |
| 1 | 2 | 6 | 8.54 | 10.52 | 5.86 | 7.84 | 10.56 | 12.65 | 9.73 ± 5.57 | 2.84 ± 4.2 | 10.42 ± 4.92 | 7.69 ± 5.99 |
| 1 | 3 | 4 | 9.24 | 18.20 | 8.26 | 14.49 | 10.13 | 21.27 | 11.88 ± 5.32 | 4.13 ± 4.79 | 11.37 ± 4.94 | 9.15 ± 6.13 |
| 1 | 3 | 5 | 8.76 | 27.93 | 7.26 | 34.78 | 10.03 | 18.72 | 12.82 ± 5.15 | 4.19 ± 5.15 | 10.98 ± 4.74 | 9.34 ± 6.23 |
| 1 | 3 | 6 | 9.56 | 17.15 | 6.51 | 14.08 | 11.85 | 19.75 | 10.44 ± 5.72 | 3 ± 4.73 | 9.86 ± 4.97 | 7.78 ± 6.16 |
| 1 | 4 | 5 | 7.99 | 8.41E+08 | 6.24 | 9.13E+08 | 9.41 | 7.62E+08 | 12.22 ± 5.45 | 4.34 ± 5.08 | 11.87 ± 4.6 | 9.49 ± 6.22 |
| 1 | 4 | 6 | 8.31 | 417.14 | 7.02 | 449.12 | 9.41 | 382.50 | 9.83 ± 5.74 | 3.15 ± 4.69 | 10.75 ± 5.03 | 7.93 ± 6.18 |
| 1 | 5 | 6 | 8.54 | 19.44 | 5.46 | 15.69 | 10.77 | 22.58 | 10.78 ± 5.92 | 3.21 ± 5.07 | 10.36 ± 4.78 | 8.13 ± 6.31 |
| 2 | 3 | 4 | 6.52 | 15.72 | 5.05 | 10.25 | 7.71 | 19.73 | 12.06 ± 5.11 | 3.08 ± 4.51 | 11.67 ± 4.91 | 8.97 ± 6.37 |
| 2 | 3 | 5 | 3.95 | 16.55 | 3.77 | 8.94 | 4.13 | 21.63 | 13.01 ± 4.9 | 3.14 ± 4.91 | 11.28 ± 4.73 | 9.16 ± 6.47 |
| 2 | 3 | 6 | 7.15 | 14.07 | 4.71 | 11.08 | 8.96 | 16.53 | 10.62 ± 5.58 | 1.94 ± 4.16 | 10.16 ± 5.02 | 7.61 ± 6.35 |
| 2 | 4 | 5 | 5.77 | 16.88 | 4.53 | 13.05 | 6.80 | 20.00 | 12.41 ± 5.24 | 3.29 ± 4.87 | 12.17 ± 4.53 | 9.31 ± 6.46 |
| 2 | 4 | 6 | 6.61 | 15.41 | 6.29 | 9.05 | 6.92 | 19.83 | 10.02 ± 5.63 | 2.1 ± 4.16 | 11.05 ± 5.04 | 7.76 ± 6.38 |
| 2 | 5 | 6 | 6.53 | 15.91 | 5.29 | 11.67 | 7.57 | 19.23 | 10.96 ± 5.78 | 2.16 ± 4.6 | 10.66 ± 4.81 | 7.95 ± 6.51 |
| 3 | 4 | 5 | 7.74 | 14.07 | 5.45 | 11.08 | 9.50 | 16.53 | 13.11 ± 5.03 | 3.44 ± 5.32 | 11.61 ± 4.79 | 9.41 ± 6.59 |
| 3 | 4 | 6 | 8.30 | 28.61 | 6.45 | 29.03 | 9.81 | 28.17 | 10.73 ± 5.74 | 2.25 ± 4.72 | 10.5 ± 5.15 | 7.86 ± 6.53 |
| 3 | 5 | 6 | 7.52 | 20.87 | 5.08 | 16.09 | 9.34 | 24.75 | 11.67 ± 5.77 | 2.31 ± 5.11 | 10.1 ± 4.89 | 8.05 ± 6.66 |
| 4 | 5 | 6 | 6.24 | 901.82 | 4.70 | 878.84 | 7.47 | 924.23 | 11.07 ± 5.92 | 2.46 ± 5.09 | 10.99 ± 4.91 | 8.2 ± 6.68 |
